# Measurement of the $b\overline{b}$ dijet cross section in *pp* collisions at $\sqrt{s} = 7$ TeV with the ATLAS detector

**DOI:** 10.1140/epjc/s10052-016-4521-y

**Published:** 2016-12-05

**Authors:** M. Aaboud, G. Aad, B. Abbott, J. Abdallah, O. Abdinov, B. Abeloos, R. Aben, O. S. AbouZeid, N. L. Abraham, H. Abramowicz, H. Abreu, R. Abreu, Y. Abulaiti, B. S. Acharya, L. Adamczyk, D. L. Adams, J. Adelman, S. Adomeit, T. Adye, A. A. Affolder, T. Agatonovic-Jovin, J. Agricola, J. A. Aguilar-Saavedra, S. P. Ahlen, F. Ahmadov, G. Aielli, H. Akerstedt, T. P. A. Åkesson, A. V. Akimov, G. L. Alberghi, J. Albert, S. Albrand, M. J. Alconada Verzini, M. Aleksa, I. N. Aleksandrov, C. Alexa, G. Alexander, T. Alexopoulos, M. Alhroob, M. Aliev, G. Alimonti, J. Alison, S. P. Alkire, B. M. M. Allbrooke, B. W. Allen, P. P. Allport, A. Aloisio, A. Alonso, F. Alonso, C. Alpigiani, M. Alstaty, B. Alvarez Gonzalez, D. Álvarez Piqueras, M. G. Alviggi, B. T. Amadio, K. Amako, Y. Amaral Coutinho, C. Amelung, D. Amidei, S. P. Amor Dos Santos, A. Amorim, S. Amoroso, G. Amundsen, C. Anastopoulos, L. S. Ancu, N. Andari, T. Andeen, C. F. Anders, G. Anders, J. K. Anders, K. J. Anderson, A. Andreazza, V. Andrei, S. Angelidakis, I. Angelozzi, P. Anger, A. Angerami, F. Anghinolfi, A. V. Anisenkov, N. Anjos, A. Annovi, M. Antonelli, A. Antonov, F. Anulli, M. Aoki, L. Aperio Bella, G. Arabidze, Y. Arai, J. P. Araque, A. T. H. Arce, F. A. Arduh, J-F. Arguin, S. Argyropoulos, M. Arik, A. J. Armbruster, L. J. Armitage, O. Arnaez, H. Arnold, M. Arratia, O. Arslan, A. Artamonov, G. Artoni, S. Artz, S. Asai, N. Asbah, A. Ashkenazi, B. Åsman, L. Asquith, K. Assamagan, R. Astalos, M. Atkinson, N. B. Atlay, K. Augsten, G. Avolio, B. Axen, M. K. Ayoub, G. Azuelos, M. A. Baak, A. E. Baas, M. J. Baca, H. Bachacou, K. Bachas, M. Backes, M. Backhaus, P. Bagiacchi, P. Bagnaia, Y. Bai, J. T. Baines, O. K. Baker, E. M. Baldin, P. Balek, T. Balestri, F. Balli, W. K. Balunas, E. Banas, Sw. Banerjee, A. A. E. Bannoura, L. Barak, E. L. Barberio, D. Barberis, M. Barbero, T. Barillari, T. Barklow, N. Barlow, S. L. Barnes, B. M. Barnett, R. M. Barnett, Z. Barnovska, A. Baroncelli, G. Barone, A. J. Barr, L. Barranco Navarro, F. Barreiro, J. Barreiro Guimarães da Costa, R. Bartoldus, A. E. Barton, P. Bartos, A. Basalaev, A. Bassalat, R. L. Bates, S. J. Batista, J. R. Batley, M. Battaglia, M. Bauce, F. Bauer, H. S. Bawa, J. B. Beacham, M. D. Beattie, T. Beau, P. H. Beauchemin, P. Bechtle, H. P. Beck, K. Becker, M. Becker, M. Beckingham, C. Becot, A. J. Beddall, A. Beddall, V. A. Bednyakov, M. Bedognetti, C. P. Bee, L. J. Beemster, T. A. Beermann, M. Begel, J. K. Behr, C. Belanger-Champagne, A. S. Bell, G. Bella, L. Bellagamba, A. Bellerive, M. Bellomo, K. Belotskiy, O. Beltramello, N. L. Belyaev, O. Benary, D. Benchekroun, M. Bender, K. Bendtz, N. Benekos, Y. Benhammou, E. Benhar Noccioli, J. Benitez, D. P. Benjamin, J. R. Bensinger, S. Bentvelsen, L. Beresford, M. Beretta, D. Berge, E. Bergeaas Kuutmann, N. Berger, J. Beringer, S. Berlendis, N. R. Bernard, C. Bernius, F. U. Bernlochner, T. Berry, P. Berta, C. Bertella, G. Bertoli, F. Bertolucci, I. A. Bertram, C. Bertsche, D. Bertsche, G. J. Besjes, O. Bessidskaia Bylund, M. Bessner, N. Besson, C. Betancourt, S. Bethke, A. J. Bevan, W. Bhimji, R. M. Bianchi, L. Bianchini, M. Bianco, O. Biebel, D. Biedermann, R. Bielski, N. V. Biesuz, M. Biglietti, J. Bilbao De Mendizabal, H. Bilokon, M. Bindi, S. Binet, A. Bingul, C. Bini, S. Biondi, D. M. Bjergaard, C. W. Black, J. E. Black, K. M. Black, D. Blackburn, R. E. Blair, J.-B. Blanchard, J. E. Blanco, T. Blazek, I. Bloch, C. Blocker, W. Blum, U. Blumenschein, S. Blunier, G. J. Bobbink, V. S. Bobrovnikov, S. S. Bocchetta, A. Bocci, C. Bock, M. Boehler, D. Boerner, J. A. Bogaerts, D. Bogavac, A. G. Bogdanchikov, C. Bohm, V. Boisvert, P. Bokan, T. Bold, A. S. Boldyrev, M. Bomben, M. Bona, M. Boonekamp, A. Borisov, G. Borissov, J. Bortfeldt, D. Bortoletto, V. Bortolotto, K. Bos, D. Boscherini, M. Bosman, J. D. Bossio Sola, J. Boudreau, J. Bouffard, E. V. Bouhova-Thacker, D. Boumediene, C. Bourdarios, S. K. Boutle, A. Boveia, J. Boyd, I. R. Boyko, J. Bracinik, A. Brandt, G. Brandt, O. Brandt, U. Bratzler, B. Brau, J. E. Brau, H. M. Braun, W. D. Breaden Madden, K. Brendlinger, A. J. Brennan, L. Brenner, R. Brenner, S. Bressler, T. M. Bristow, D. Britton, D. Britzger, F. M. Brochu, I. Brock, R. Brock, G. Brooijmans, T. Brooks, W. K. Brooks, J. Brosamer, E. Brost, J. H Broughton, P. A. Bruckman de Renstrom, D. Bruncko, R. Bruneliere, A. Bruni, G. Bruni, L. S. Bruni, BH Brunt, M. Bruschi, N. Bruscino, P. Bryant, L. Bryngemark, T. Buanes, Q. Buat, P. Buchholz, A. G. Buckley, I. A. Budagov, F. Buehrer, M. K. Bugge, O. Bulekov, D. Bullock, H. Burckhart, S. Burdin, C. D. Burgard, B. Burghgrave, K. Burka, S. Burke, I. Burmeister, E. Busato, D. Büscher, V. Büscher, P. Bussey, J. M. Butler, C. M. Buttar, J. M. Butterworth, P. Butti, W. Buttinger, A. Buzatu, A. R. Buzykaev, S. Cabrera Urbán, D. Caforio, V. M. Cairo, O. Cakir, N. Calace, P. Calafiura, A. Calandri, G. Calderini, P. Calfayan, L. P. Caloba, D. Calvet, S. Calvet, T. P. Calvet, R. Camacho Toro, S. Camarda, P. Camarri, D. Cameron, R. Caminal Armadans, C. Camincher, S. Campana, M. Campanelli, A. Camplani, A. Campoverde, V. Canale, A. Canepa, M. Cano Bret, J. Cantero, R. Cantrill, T. Cao, M. D. M. Capeans Garrido, I. Caprini, M. Caprini, M. Capua, R. Caputo, R. M. Carbone, R. Cardarelli, F. Cardillo, I. Carli, T. Carli, G. Carlino, L. Carminati, S. Caron, E. Carquin, G. D. Carrillo-Montoya, J. R. Carter, J. Carvalho, D. Casadei, M. P. Casado, M. Casolino, D. W. Casper, E. Castaneda-Miranda, R. Castelijn, A. Castelli, V. Castillo Gimenez, N. F. Castro, A. Catinaccio, J. R. Catmore, A. Cattai, J. Caudron, V. Cavaliere, E. Cavallaro, D. Cavalli, M. Cavalli-Sforza, V. Cavasinni, F. Ceradini, L. Cerda Alberich, B. C. Cerio, A. S. Cerqueira, A. Cerri, L. Cerrito, F. Cerutti, M. Cerv, A. Cervelli, S. A. Cetin, A. Chafaq, D. Chakraborty, S. K. Chan, Y. L. Chan, P. Chang, J. D. Chapman, D. G. Charlton, A. Chatterjee, C. C. Chau, C. A. Chavez Barajas, S. Che, S. Cheatham, A. Chegwidden, S. Chekanov, S. V. Chekulaev, G. A. Chelkov, M. A. Chelstowska, C. Chen, H. Chen, K. Chen, S. Chen, S. Chen, X. Chen, Y. Chen, H. C. Cheng, H. J Cheng, Y. Cheng, A. Cheplakov, E. Cheremushkina, R. Cherkaoui El Moursli, V. Chernyatin, E. Cheu, L. Chevalier, V. Chiarella, G. Chiarelli, G. Chiodini, A. S. Chisholm, A. Chitan, M. V. Chizhov, K. Choi, A. R. Chomont, S. Chouridou, B. K. B. Chow, V. Christodoulou, D. Chromek-Burckhart, J. Chudoba, A. J. Chuinard, J. J. Chwastowski, L. Chytka, G. Ciapetti, A. K. Ciftci, D. Cinca, V. Cindro, I. A. Cioara, A. Ciocio, F. Cirotto, Z. H. Citron, M. Citterio, M. Ciubancan, A. Clark, B. L. Clark, M. R. Clark, P. J. Clark, R. N. Clarke, C. Clement, Y. Coadou, M. Cobal, A. Coccaro, J. Cochran, L. Coffey, L. Colasurdo, B. Cole, A. P. Colijn, J. Collot, T. Colombo, G. Compostella, P. Conde Muiño, E. Coniavitis, S. H. Connell, I. A. Connelly, V. Consorti, S. Constantinescu, G. Conti, F. Conventi, M. Cooke, B. D. Cooper, A. M. Cooper-Sarkar, K. J. R. Cormier, T. Cornelissen, M. Corradi, F. Corriveau, A. Corso-Radu, A. Cortes-Gonzalez, G. Cortiana, G. Costa, M. J. Costa, D. Costanzo, G. Cottin, G. Cowan, B. E. Cox, K. Cranmer, S. J. Crawley, G. Cree, S. Crépé-Renaudin, F. Crescioli, W. A. Cribbs, M. Crispin Ortuzar, M. Cristinziani, V. Croft, G. Crosetti, T. Cuhadar Donszelmann, J. Cummings, M. Curatolo, J. Cúth, C. Cuthbert, H. Czirr, P. Czodrowski, G. D’amen, S. D’Auria, M. D’Onofrio, M. J. Da Cunha Sargedas De Sousa, C. Da Via, W. Dabrowski, T. Dado, T. Dai, O. Dale, F. Dallaire, C. Dallapiccola, M. Dam, J. R. Dandoy, N. P. Dang, A. C. Daniells, N. S. Dann, M. Danninger, M. Dano Hoffmann, V. Dao, G. Darbo, S. Darmora, J. Dassoulas, A. Dattagupta, W. Davey, C. David, T. Davidek, M. Davies, P. Davison, E. Dawe, I. Dawson, R. K. Daya-Ishmukhametova, K. De, R. de Asmundis, A. De Benedetti, S. De Castro, S. De Cecco, N. De Groot, P. de Jong, H. De la Torre, F. De Lorenzi, A. De Maria, D. De Pedis, A. De Salvo, U. De Sanctis, A. De Santo, J. B. De Vivie De Regie, W. J. Dearnaley, R. Debbe, C. Debenedetti, D. V. Dedovich, N. Dehghanian, I. Deigaard, M. Del Gaudio, J. Del Peso, T. Del Prete, D. Delgove, F. Deliot, C. M. Delitzsch, M. Deliyergiyev, A. Dell’Acqua, L. Dell’Asta, M. Dell’Orso, M. Della Pietra, D. della Volpe, M. Delmastro, P. A. Delsart, C. Deluca, D. A. DeMarco, S. Demers, M. Demichev, A. Demilly, S. P. Denisov, D. Denysiuk, D. Derendarz, J. E. Derkaoui, F. Derue, P. Dervan, K. Desch, C. Deterre, K. Dette, P. O. Deviveiros, A. Dewhurst, S. Dhaliwal, A. Di Ciaccio, L. Di Ciaccio, W. K. Di Clemente, C. Di Donato, A. Di Girolamo, B. Di Girolamo, B. Di Micco, R. Di Nardo, A. Di Simone, R. Di Sipio, D. Di Valentino, C. Diaconu, M. Diamond, F. A. Dias, M. A. Diaz, E. B. Diehl, J. Dietrich, S. Diglio, A. Dimitrievska, J. Dingfelder, P. Dita, S. Dita, F. Dittus, F. Djama, T. Djobava, J. I. Djuvsland, M. A. B. do Vale, D. Dobos, M. Dobre, C. Doglioni, T. Dohmae, J. Dolejsi, Z. Dolezal, B. A. Dolgoshein, M. Donadelli, S. Donati, P. Dondero, J. Donini, J. Dopke, A. Doria, M. T. Dova, A. T. Doyle, E. Drechsler, M. Dris, Y. Du, J. Duarte-Campderros, E. Duchovni, G. Duckeck, O. A. Ducu, D. Duda, A. Dudarev, E. M. Duffield, L. Duflot, L. Duguid, M. Dührssen, M. Dumancic, M. Dunford, H. Duran Yildiz, M. Düren, A. Durglishvili, D. Duschinger, B. Dutta, M. Dyndal, C. Eckardt, K. M. Ecker, R. C. Edgar, N. C. Edwards, T. Eifert, G. Eigen, K. Einsweiler, T. Ekelof, M. El Kacimi, V. Ellajosyula, M. Ellert, S. Elles, F. Ellinghaus, A. A. Elliot, N. Ellis, J. Elmsheuser, M. Elsing, D. Emeliyanov, Y. Enari, O. C. Endner, M. Endo, J. S. Ennis, J. Erdmann, A. Ereditato, G. Ernis, J. Ernst, M. Ernst, S. Errede, E. Ertel, M. Escalier, H. Esch, C. Escobar, B. Esposito, A. I. Etienvre, E. Etzion, H. Evans, A. Ezhilov, F. Fabbri, L. Fabbri, G. Facini, R. M. Fakhrutdinov, S. Falciano, R. J. Falla, J. Faltova, Y. Fang, M. Fanti, A. Farbin, A. Farilla, C. Farina, T. Farooque, S. Farrell, S. M. Farrington, P. Farthouat, F. Fassi, P. Fassnacht, D. Fassouliotis, M. Faucci Giannelli, A. Favareto, W. J. Fawcett, L. Fayard, O. L. Fedin, W. Fedorko, S. Feigl, L. Feligioni, C. Feng, E. J. Feng, H. Feng, A. B. Fenyuk, L. Feremenga, P. Fernandez Martinez, S. Fernandez Perez, J. Ferrando, A. Ferrari, P. Ferrari, R. Ferrari, D. E. Ferreira de Lima, A. Ferrer, D. Ferrere, C. Ferretti, A. Ferretto Parodi, F. Fiedler, A. Filipčič, M. Filipuzzi, F. Filthaut, M. Fincke-Keeler, K. D. Finelli, M. C. N. Fiolhais, L. Fiorini, A. Firan, A. Fischer, C. Fischer, J. Fischer, W. C. Fisher, N. Flaschel, I. Fleck, P. Fleischmann, G. T. Fletcher, R. R. M. Fletcher, T. Flick, A. Floderus, L. R. Flores Castillo, M. J. Flowerdew, G. T. Forcolin, A. Formica, A. Forti, A. G. Foster, D. Fournier, H. Fox, S. Fracchia, P. Francavilla, M. Franchini, D. Francis, L. Franconi, M. Franklin, M. Frate, M. Fraternali, D. Freeborn, S. M. Fressard-Batraneanu, F. Friedrich, D. Froidevaux, J. A. Frost, C. Fukunaga, E. Fullana Torregrosa, T. Fusayasu, J. Fuster, C. Gabaldon, O. Gabizon, A. Gabrielli, A. Gabrielli, G. P. Gach, S. Gadatsch, S. Gadomski, G. Gagliardi, L. G. Gagnon, P. Gagnon, C. Galea, B. Galhardo, E. J. Gallas, B. J. Gallop, P. Gallus, G. Galster, K. K. Gan, J. Gao, Y. Gao, Y. S. Gao, F. M. Garay Walls, C. García, J. E. García Navarro, M. Garcia-Sciveres, R. W. Gardner, N. Garelli, V. Garonne, A. Gascon Bravo, C. Gatti, A. Gaudiello, G. Gaudio, B. Gaur, L. Gauthier, I. L. Gavrilenko, C. Gay, G. Gaycken, E. N. Gazis, Z. Gecse, C. N. P. Gee, Ch. Geich-Gimbel, M. Geisen, M. P. Geisler, C. Gemme, M. H. Genest, C. Geng, S. Gentile, S. George, D. Gerbaudo, A. Gershon, S. Ghasemi, H. Ghazlane, M. Ghneimat, B. Giacobbe, S. Giagu, P. Giannetti, B. Gibbard, S. M. Gibson, M. Gignac, M. Gilchriese, T. P. S. Gillam, D. Gillberg, G. Gilles, D. M. Gingrich, N. Giokaris, M. P. Giordani, F. M. Giorgi, F. M. Giorgi, P. F. Giraud, P. Giromini, D. Giugni, F. Giuli, C. Giuliani, M. Giulini, B. K. Gjelsten, S. Gkaitatzis, I. Gkialas, E. L. Gkougkousis, L. K. Gladilin, C. Glasman, J. Glatzer, P. C. F. Glaysher, A. Glazov, M. Goblirsch-Kolb, J. Godlewski, S. Goldfarb, T. Golling, D. Golubkov, A. Gomes, R. Gonçalo, J. Goncalves Pinto Firmino Da Costa, G. Gonella, L. Gonella, A. Gongadze, S. González de la Hoz, G. Gonzalez Parra, S. Gonzalez-Sevilla, L. Goossens, P. A. Gorbounov, H. A. Gordon, I. Gorelov, B. Gorini, E. Gorini, A. Gorišek, E. Gornicki, A. T. Goshaw, C. Gössling, M. I. Gostkin, C. R. Goudet, D. Goujdami, A. G. Goussiou, N. Govender, E. Gozani, L. Graber, I. Grabowska-Bold, P. O. J. Gradin, P. Grafström, J. Gramling, E. Gramstad, S. Grancagnolo, V. Gratchev, P. M. Gravila, H. M. Gray, E. Graziani, Z. D. Greenwood, C. Grefe, K. Gregersen, I. M. Gregor, P. Grenier, K. Grevtsov, J. Griffiths, A. A. Grillo, K. Grimm, S. Grinstein, Ph. Gris, J.-F. Grivaz, S. Groh, J. P. Grohs, E. Gross, J. Grosse-Knetter, G. C. Grossi, Z. J. Grout, L. Guan, W. Guan, J. Guenther, F. Guescini, D. Guest, O. Gueta, E. Guido, T. Guillemin, S. Guindon, U. Gul, C. Gumpert, J. Guo, Y. Guo, S. Gupta, G. Gustavino, P. Gutierrez, N. G. Gutierrez Ortiz, C. Gutschow, C. Guyot, C. Gwenlan, C. B. Gwilliam, A. Haas, C. Haber, H. K. Hadavand, N. Haddad, A. Hadef, P. Haefner, S. Hageböck, Z. Hajduk, H. Hakobyan, M. Haleem, J. Haley, G. Halladjian, G. D. Hallewell, K. Hamacher, P. Hamal, K. Hamano, A. Hamilton, G. N. Hamity, P. G. Hamnett, L. Han, K. Hanagaki, K. Hanawa, M. Hance, B. Haney, P. Hanke, R. Hanna, J. B. Hansen, J. D. Hansen, M. C. Hansen, P. H. Hansen, K. Hara, A. S. Hard, T. Harenberg, F. Hariri, S. Harkusha, R. D. Harrington, P. F. Harrison, F. Hartjes, N. M. Hartmann, M. Hasegawa, Y. Hasegawa, A. Hasib, S. Hassani, S. Haug, R. Hauser, L. Hauswald, M. Havranek, C. M. Hawkes, R. J. Hawkings, D. Hayden, C. P. Hays, J. M. Hays, H. S. Hayward, S. J. Haywood, S. J. Head, T. Heck, V. Hedberg, L. Heelan, S. Heim, T. Heim, B. Heinemann, J. J. Heinrich, L. Heinrich, C. Heinz, J. Hejbal, L. Helary, S. Hellman, C. Helsens, J. Henderson, R. C. W. Henderson, Y. Heng, S. Henkelmann, A. M. Henriques Correia, S. Henrot-Versille, G. H. Herbert, Y. Hernández Jiménez, G. Herten, R. Hertenberger, L. Hervas, G. G. Hesketh, N. P. Hessey, J. W. Hetherly, R. Hickling, E. Higón-Rodriguez, E. Hill, J. C. Hill, K. H. Hiller, S. J. Hillier, I. Hinchliffe, E. Hines, R. R. Hinman, M. Hirose, D. Hirschbuehl, J. Hobbs, N. Hod, M. C. Hodgkinson, P. Hodgson, A. Hoecker, M. R. Hoeferkamp, F. Hoenig, D. Hohn, T. R. Holmes, M. Homann, T. M. Hong, B. H. Hooberman, W. H. Hopkins, Y. Horii, A. J. Horton, J-Y. Hostachy, S. Hou, A. Hoummada, J. Howarth, M. Hrabovsky, I. Hristova, J. Hrivnac, T. Hryn’ova, A. Hrynevich, C. Hsu, P. J. Hsu, S.-C. Hsu, D. Hu, Q. Hu, Y. Huang, Z. Hubacek, F. Hubaut, F. Huegging, T. B. Huffman, E. W. Hughes, G. Hughes, M. Huhtinen, T. A. Hülsing, P. Huo, N. Huseynov, J. Huston, J. Huth, G. Iacobucci, G. Iakovidis, I. Ibragimov, L. Iconomidou-Fayard, E. Ideal, Z. Idrissi, P. Iengo, O. Igonkina, T. Iizawa, Y. Ikegami, M. Ikeno, Y. Ilchenko, D. Iliadis, N. Ilic, T. Ince, G. Introzzi, P. Ioannou, M. Iodice, K. Iordanidou, V. Ippolito, M. Ishino, M. Ishitsuka, R. Ishmukhametov, C. Issever, S. Istin, F. Ito, J. M. Iturbe Ponce, R. Iuppa, W. Iwanski, H. Iwasaki, J. M. Izen, V. Izzo, S. Jabbar, B. Jackson, M. Jackson, P. Jackson, V. Jain, K. B. Jakobi, K. Jakobs, S. Jakobsen, T. Jakoubek, D. O. Jamin, D. K. Jana, E. Jansen, R. Jansky, J. Janssen, M. Janus, G. Jarlskog, N. Javadov, T. Javůrek, F. Jeanneau, L. Jeanty, J. Jejelava, G.-Y. Jeng, D. Jennens, P. Jenni, J. Jentzsch, C. Jeske, S. Jézéquel, H. Ji, J. Jia, H. Jiang, Y. Jiang, S. Jiggins, J. Jimenez Pena, S. Jin, A. Jinaru, O. Jinnouchi, P. Johansson, K. A. Johns, W. J. Johnson, K. Jon-And, G. Jones, R. W. L. Jones, S. Jones, T. J. Jones, J. Jongmanns, P. M. Jorge, J. Jovicevic, X. Ju, A. Juste Rozas, M. K. Köhler, A. Kaczmarska, M. Kado, H. Kagan, M. Kagan, S. J. Kahn, E. Kajomovitz, C. W. Kalderon, A. Kaluza, S. Kama, A. Kamenshchikov, N. Kanaya, S. Kaneti, L. Kanjir, V. A. Kantserov, J. Kanzaki, B. Kaplan, L. S. Kaplan, A. Kapliy, D. Kar, K. Karakostas, A. Karamaoun, N. Karastathis, M. J. Kareem, E. Karentzos, M. Karnevskiy, S. N. Karpov, Z. M. Karpova, K. Karthik, V. Kartvelishvili, A. N. Karyukhin, K. Kasahara, L. Kashif, R. D. Kass, A. Kastanas, Y. Kataoka, C. Kato, A. Katre, J. Katzy, K. Kawagoe, T. Kawamoto, G. Kawamura, S. Kazama, V. F. Kazanin, R. Keeler, R. Kehoe, J. S. Keller, J. J. Kempster, K Kentaro, H. Keoshkerian, O. Kepka, B. P. Kerševan, S. Kersten, R. A. Keyes, F. Khalil-zada, A. Khanov, A. G. Kharlamov, T. J. Khoo, V. Khovanskiy, E. Khramov, J. Khubua, S. Kido, H. Y. Kim, S. H. Kim, Y. K. Kim, N. Kimura, O. M. Kind, B. T. King, M. King, S. B. King, J. Kirk, A. E. Kiryunin, T. Kishimoto, D. Kisielewska, F. Kiss, K. Kiuchi, O. Kivernyk, E. Kladiva, M. H. Klein, M. Klein, U. Klein, K. Kleinknecht, P. Klimek, A. Klimentov, R. Klingenberg, J. A. Klinger, T. Klioutchnikova, E.-E. Kluge, P. Kluit, S. Kluth, J. Knapik, E. Kneringer, E. B. F. G. Knoops, A. Knue, A. Kobayashi, D. Kobayashi, T. Kobayashi, M. Kobel, M. Kocian, P. Kodys, T. Koffas, E. Koffeman, T. Koi, H. Kolanoski, M. Kolb, I. Koletsou, A. A. Komar, Y. Komori, T. Kondo, N. Kondrashova, K. Köneke, A. C. König, T. Kono, R. Konoplich, N. Konstantinidis, R. Kopeliansky, S. Koperny, L. Köpke, A. K. Kopp, K. Korcyl, K. Kordas, A. Korn, A. A. Korol, I. Korolkov, E. V. Korolkova, O. Kortner, S. Kortner, T. Kosek, V. V. Kostyukhin, A. Kotwal, A. Kourkoumeli-Charalampidi, C. Kourkoumelis, V. Kouskoura, A. B. Kowalewska, R. Kowalewski, T. Z. Kowalski, C. Kozakai, W. Kozanecki, A. S. Kozhin, V. A. Kramarenko, G. Kramberger, D. Krasnopevtsev, M. W. Krasny, A. Krasznahorkay, J. K. Kraus, A. Kravchenko, M. Kretz, J. Kretzschmar, K. Kreutzfeldt, P. Krieger, K. Krizka, K. Kroeninger, H. Kroha, J. Kroll, J. Kroseberg, J. Krstic, U. Kruchonak, H. Krüger, N. Krumnack, A. Kruse, M. C. Kruse, M. Kruskal, T. Kubota, H. Kucuk, S. Kuday, J. T. Kuechler, S. Kuehn, A. Kugel, F. Kuger, A. Kuhl, T. Kuhl, V. Kukhtin, R. Kukla, Y. Kulchitsky, S. Kuleshov, M. Kuna, T. Kunigo, A. Kupco, H. Kurashige, Y. A. Kurochkin, V. Kus, E. S. Kuwertz, M. Kuze, J. Kvita, T. Kwan, D. Kyriazopoulos, A. La Rosa, J. L. La Rosa Navarro, L. La Rotonda, C. Lacasta, F. Lacava, J. Lacey, H. Lacker, D. Lacour, V. R. Lacuesta, E. Ladygin, R. Lafaye, B. Laforge, T. Lagouri, S. Lai, S. Lammers, W. Lampl, E. Lançon, U. Landgraf, M. P. J. Landon, V. S. Lang, J. C. Lange, A. J. Lankford, F. Lanni, K. Lantzsch, A. Lanza, S. Laplace, C. Lapoire, J. F. Laporte, T. Lari, F. Lasagni Manghi, M. Lassnig, P. Laurelli, W. Lavrijsen, A. T. Law, P. Laycock, T. Lazovich, M. Lazzaroni, B. Le, O. Le Dortz, E. Le Guirriec, E. P. Le Quilleuc, M. LeBlanc, T. LeCompte, F. Ledroit-Guillon, C. A. Lee, S. C. Lee, L. Lee, G. Lefebvre, M. Lefebvre, F. Legger, C. Leggett, A. Lehan, G. Lehmann Miotto, X. Lei, W. A. Leight, A. Leisos, A. G. Leister, M. A. L. Leite, R. Leitner, D. Lellouch, B. Lemmer, K. J. C. Leney, T. Lenz, B. Lenzi, R. Leone, S. Leone, C. Leonidopoulos, S. Leontsinis, G. Lerner, C. Leroy, A. A. J. Lesage, C. G. Lester, M. Levchenko, J. Levêque, D. Levin, L. J. Levinson, M. Levy, D. Lewis, A. M. Leyko, M. Leyton, B. Li, H. Li, H. L. Li, L. Li, L. Li, Q. Li, S. Li, X. Li, Y. Li, Z. Liang, B. Liberti, A. Liblong, P. Lichard, K. Lie, J. Liebal, W. Liebig, A. Limosani, S. C. Lin, T. H. Lin, B. E. Lindquist, A. E. Lionti, E. Lipeles, A. Lipniacka, M. Lisovyi, T. M. Liss, A. Lister, A. M. Litke, B. Liu, D. Liu, H. Liu, H. Liu, J. Liu, J. B. Liu, K. Liu, L. Liu, M. Liu, M. Liu, Y. L. Liu, Y. Liu, M. Livan, A. Lleres, J. Llorente Merino, S. L. Lloyd, F. Lo Sterzo, E. Lobodzinska, P. Loch, W. S. Lockman, F. K. Loebinger, A. E. Loevschall-Jensen, K. M. Loew, A. Loginov, T. Lohse, K. Lohwasser, M. Lokajicek, B. A. Long, J. D. Long, R. E. Long, L. Longo, K. A. Looper, L. Lopes, D. Lopez Mateos, B. Lopez Paredes, I. Lopez Paz, A. Lopez Solis, J. Lorenz, N. Lorenzo Martinez, M. Losada, P. J. Lösel, X. Lou, A. Lounis, J. Love, P. A. Love, H. Lu, N. Lu, H. J. Lubatti, C. Luci, A. Lucotte, C. Luedtke, F. Luehring, W. Lukas, L. Luminari, O. Lundberg, B. Lund-Jensen, P. M. Luzi, D. Lynn, R. Lysak, E. Lytken, V. Lyubushkin, H. Ma, L. L. Ma, Y. Ma, G. Maccarrone, A. Macchiolo, C. M. Macdonald, B. Maček, J. Machado Miguens, D. Madaffari, R. Madar, H. J. Maddocks, W. F. Mader, A. Madsen, J. Maeda, S. Maeland, T. Maeno, A. Maevskiy, E. Magradze, J. Mahlstedt, C. Maiani, C. Maidantchik, A. A. Maier, T. Maier, A. Maio, S. Majewski, Y. Makida, N. Makovec, B. Malaescu, Pa. Malecki, V. P. Maleev, F. Malek, U. Mallik, D. Malon, C. Malone, S. Maltezos, S. Malyukov, J. Mamuzic, G. Mancini, B. Mandelli, L. Mandelli, I. Mandić, J. Maneira, L. Manhaes de Andrade Filho, J. Manjarres Ramos, A. Mann, A. Manousos, B. Mansoulie, J. D. Mansour, R. Mantifel, M. Mantoani, S. Manzoni, L. Mapelli, G. Marceca, L. March, G. Marchiori, M. Marcisovsky, M. Marjanovic, D. E. Marley, F. Marroquim, S. P. Marsden, Z. Marshall, S. Marti-Garcia, B. Martin, T. A. Martin, V. J. Martin, B. Martin dit Latour, M. Martinez, S. Martin-Haugh, V. S. Martoiu, A. C. Martyniuk, M. Marx, A. Marzin, L. Masetti, T. Mashimo, R. Mashinistov, J. Masik, A. L. Maslennikov, I. Massa, L. Massa, P. Mastrandrea, A. Mastroberardino, T. Masubuchi, P. Mättig, J. Mattmann, J. Maurer, S. J. Maxfield, D. A. Maximov, R. Mazini, S. M. Mazza, N. C. Mc Fadden, G. Mc Goldrick, S. P. Mc Kee, A. McCarn, R. L. McCarthy, T. G. McCarthy, L. I. McClymont, E. F. McDonald, K. W. McFarlane, J. A. Mcfayden, G. Mchedlidze, S. J. McMahon, R. A. McPherson, M. Medinnis, S. Meehan, S. Mehlhase, A. Mehta, K. Meier, C. Meineck, B. Meirose, D. Melini, B. R. Mellado Garcia, M. Melo, F. Meloni, A. Mengarelli, S. Menke, E. Meoni, S. Mergelmeyer, P. Mermod, L. Merola, C. Meroni, F. S. Merritt, A. Messina, J. Metcalfe, A. S. Mete, C. Meyer, C. Meyer, J-P. Meyer, J. Meyer, H. Meyer Zu Theenhausen, F. Miano, R. P. Middleton, S. Miglioranzi, L. Mijović, G. Mikenberg, M. Mikestikova, M. Mikuž, M. Milesi, A. Milic, D. W. Miller, C. Mills, A. Milov, D. A. Milstead, A. A. Minaenko, Y. Minami, I. A. Minashvili, A. I. Mincer, B. Mindur, M. Mineev, Y. Ming, L. M. Mir, K. P. Mistry, T. Mitani, J. Mitrevski, V. A. Mitsou, A. Miucci, P. S. Miyagawa, J. U. Mjörnmark, T. Moa, K. Mochizuki, S. Mohapatra, S. Molander, R. Moles-Valls, R. Monden, M. C. Mondragon, K. Mönig, J. Monk, E. Monnier, A. Montalbano, J. Montejo Berlingen, F. Monticelli, S. Monzani, R. W. Moore, N. Morange, D. Moreno, M. Moreno Llácer, P. Morettini, D. Mori, T. Mori, M. Morii, M. Morinaga, V. Morisbak, S. Moritz, A. K. Morley, G. Mornacchi, J. D. Morris, S. S. Mortensen, L. Morvaj, M. Mosidze, J. Moss, K. Motohashi, R. Mount, E. Mountricha, S. V. Mouraviev, E. J. W. Moyse, S. Muanza, R. D. Mudd, F. Mueller, J. Mueller, R. S. P. Mueller, T. Mueller, D. Muenstermann, P. Mullen, G. A. Mullier, F. J. Munoz Sanchez, J. A. Murillo Quijada, W. J. Murray, H. Musheghyan, M. Muškinja, A. G. Myagkov, M. Myska, B. P. Nachman, O. Nackenhorst, K. Nagai, R. Nagai, K. Nagano, Y. Nagasaka, K. Nagata, M. Nagel, E. Nagy, A. M. Nairz, Y. Nakahama, K. Nakamura, T. Nakamura, I. Nakano, H. Namasivayam, R. F. Naranjo Garcia, R. Narayan, D. I. Narrias Villar, I. Naryshkin, T. Naumann, G. Navarro, R. Nayyar, H. A. Neal, P. Yu. Nechaeva, T. J. Neep, P. D. Nef, A. Negri, M. Negrini, S. Nektarijevic, C. Nellist, A. Nelson, S. Nemecek, P. Nemethy, A. A. Nepomuceno, M. Nessi, M. S. Neubauer, M. Neumann, R. M. Neves, P. Nevski, P. R. Newman, D. H. Nguyen, T. Nguyen Manh, R. B. Nickerson, R. Nicolaidou, J. Nielsen, A. Nikiforov, V. Nikolaenko, I. Nikolic-Audit, K. Nikolopoulos, J. K. Nilsen, P. Nilsson, Y. Ninomiya, A. Nisati, R. Nisius, T. Nobe, L. Nodulman, M. Nomachi, I. Nomidis, T. Nooney, S. Norberg, M. Nordberg, N. Norjoharuddeen, O. Novgorodova, S. Nowak, M. Nozaki, L. Nozka, K. Ntekas, E. Nurse, F. Nuti, F. O’grady, D. C. O’Neil, A. A. O’Rourke, V. O’Shea, F. G. Oakham, H. Oberlack, T. Obermann, J. Ocariz, A. Ochi, I. Ochoa, J. P. Ochoa-Ricoux, S. Oda, S. Odaka, H. Ogren, A. Oh, S. H. Oh, C. C. Ohm, H. Ohman, H. Oide, H. Okawa, Y. Okumura, T. Okuyama, A. Olariu, L. F. Oleiro Seabra, S. A. Olivares Pino, D. Oliveira Damazio, A. Olszewski, J. Olszowska, A. Onofre, K. Onogi, P. U. E. Onyisi, M. J. Oreglia, Y. Oren, D. Orestano, N. Orlando, R. S. Orr, B. Osculati, R. Ospanov, G. Otero y Garzon, H. Otono, M. Ouchrif, F. Ould-Saada, A. Ouraou, K. P. Oussoren, Q. Ouyang, M. Owen, R. E. Owen, V. E. Ozcan, N. Ozturk, K. Pachal, A. Pacheco Pages, L. Pacheco Rodriguez, C. Padilla Aranda, M. Pagáčová, S. Pagan Griso, F. Paige, P. Pais, K. Pajchel, G. Palacino, S. Palestini, M. Palka, D. Pallin, A. Palma, E. St. Panagiotopoulou, C. E. Pandini, J. G. Panduro Vazquez, P. Pani, S. Panitkin, D. Pantea, L. Paolozzi, Th. D. Papadopoulou, K. Papageorgiou, A. Paramonov, D. Paredes Hernandez, A. J. Parker, M. A. Parker, K. A. Parker, F. Parodi, J. A. Parsons, U. Parzefall, V. R. Pascuzzi, E. Pasqualucci, S. Passaggio, Fr. Pastore, G. Pásztor, S. Pataraia, J. R. Pater, T. Pauly, J. Pearce, B. Pearson, L. E. Pedersen, M. Pedersen, S. Pedraza Lopez, R. Pedro, S. V. Peleganchuk, D. Pelikan, O. Penc, C. Peng, H. Peng, J. Penwell, B. S. Peralva, M. M. Perego, D. V. Perepelitsa, E. Perez Codina, L. Perini, H. Pernegger, S. Perrella, R. Peschke, V. D. Peshekhonov, K. Peters, R. F. Y. Peters, B. A. Petersen, T. C. Petersen, E. Petit, A. Petridis, C. Petridou, P. Petroff, E. Petrolo, M. Petrov, F. Petrucci, N. E. Pettersson, A. Peyaud, R. Pezoa, P. W. Phillips, G. Piacquadio, E. Pianori, A. Picazio, E. Piccaro, M. Piccinini, M. A. Pickering, R. Piegaia, J. E. Pilcher, A. D. Pilkington, A. W. J. Pin, M. Pinamonti, J. L. Pinfold, A. Pingel, S. Pires, H. Pirumov, M. Pitt, L. Plazak, M.-A. Pleier, V. Pleskot, E. Plotnikova, P. Plucinski, D. Pluth, R. Poettgen, L. Poggioli, D. Pohl, G. Polesello, A. Poley, A. Policicchio, R. Polifka, A. Polini, C. S. Pollard, V. Polychronakos, K. Pommès, L. Pontecorvo, B. G. Pope, G. A. Popeneciu, D. S. Popovic, A. Poppleton, S. Pospisil, K. Potamianos, I. N. Potrap, C. J. Potter, C. T. Potter, G. Poulard, J. Poveda, V. Pozdnyakov, M. E. Pozo Astigarraga, P. Pralavorio, A. Pranko, S. Prell, D. Price, L. E. Price, M. Primavera, S. Prince, M. Proissl, K. Prokofiev, F. Prokoshin, S. Protopopescu, J. Proudfoot, M. Przybycien, D. Puddu, M. Purohit, P. Puzo, J. Qian, G. Qin, Y. Qin, A. Quadt, W. B. Quayle, M. Queitsch-Maitland, D. Quilty, S. Raddum, V. Radeka, V. Radescu, S. K. Radhakrishnan, P. Radloff, P. Rados, F. Ragusa, G. Rahal, J. A. Raine, S. Rajagopalan, M. Rammensee, C. Rangel-Smith, M. G. Ratti, F. Rauscher, S. Rave, T. Ravenscroft, I. Ravinovich, M. Raymond, A. L. Read, N. P. Readioff, M. Reale, D. M. Rebuzzi, A. Redelbach, G. Redlinger, R. Reece, K. Reeves, L. Rehnisch, J. Reichert, H. Reisin, C. Rembser, H. Ren, M. Rescigno, S. Resconi, O. L. Rezanova, P. Reznicek, R. Rezvani, R. Richter, S. Richter, E. Richter-Was, O. Ricken, M. Ridel, P. Rieck, C. J. Riegel, J. Rieger, O. Rifki, M. Rijssenbeek, A. Rimoldi, M. Rimoldi, L. Rinaldi, B. Ristić, E. Ritsch, I. Riu, F. Rizatdinova, E. Rizvi, C. Rizzi, S. H. Robertson, A. Robichaud-Veronneau, D. Robinson, J. E. M. Robinson, A. Robson, C. Roda, Y. Rodina, A. Rodriguez Perez, D. Rodriguez Rodriguez, S. Roe, C. S. Rogan, O. Røhne, A. Romaniouk, M. Romano, S. M. Romano Saez, E. Romero Adam, N. Rompotis, M. Ronzani, L. Roos, E. Ros, S. Rosati, K. Rosbach, P. Rose, O. Rosenthal, N.-A. Rosien, V. Rossetti, E. Rossi, L. P. Rossi, J. H. N. Rosten, R. Rosten, M. Rotaru, I. Roth, J. Rothberg, D. Rousseau, C. R. Royon, A. Rozanov, Y. Rozen, X. Ruan, F. Rubbo, M. S. Rudolph, F. Rühr, A. Ruiz-Martinez, Z. Rurikova, N. A. Rusakovich, A. Ruschke, H. L. Russell, J. P. Rutherfoord, N. Ruthmann, Y. F. Ryabov, M. Rybar, G. Rybkin, S. Ryu, A. Ryzhov, G. F. Rzehorz, A. F. Saavedra, G. Sabato, S. Sacerdoti, H. F-W. Sadrozinski, R. Sadykov, F. Safai Tehrani, P. Saha, M. Sahinsoy, M. Saimpert, T. Saito, H. Sakamoto, Y. Sakurai, G. Salamanna, A. Salamon, J. E. Salazar Loyola, D. Salek, P. H. Sales De Bruin, D. Salihagic, A. Salnikov, J. Salt, D. Salvatore, F. Salvatore, A. Salvucci, A. Salzburger, D. Sammel, D. Sampsonidis, A. Sanchez, J. Sánchez, V. Sanchez Martinez, H. Sandaker, R. L. Sandbach, H. G. Sander, M. Sandhoff, C. Sandoval, R. Sandstroem, D. P. C. Sankey, M. Sannino, A. Sansoni, C. Santoni, R. Santonico, H. Santos, I. Santoyo Castillo, K. Sapp, A. Sapronov, J. G. Saraiva, B. Sarrazin, O. Sasaki, Y. Sasaki, K. Sato, G. Sauvage, E. Sauvan, G. Savage, P. Savard, C. Sawyer, L. Sawyer, J. Saxon, C. Sbarra, A. Sbrizzi, T. Scanlon, D. A. Scannicchio, M. Scarcella, V. Scarfone, J. Schaarschmidt, P. Schacht, B. M. Schachtner, D. Schaefer, R. Schaefer, J. Schaeffer, S. Schaepe, S. Schaetzel, U. Schäfer, A. C. Schaffer, D. Schaile, R. D. Schamberger, V. Scharf, V. A. Schegelsky, D. Scheirich, M. Schernau, C. Schiavi, S. Schier, C. Schillo, M. Schioppa, S. Schlenker, K. R. Schmidt-Sommerfeld, K. Schmieden, C. Schmitt, S. Schmitt, S. Schmitz, B. Schneider, U. Schnoor, L. Schoeffel, A. Schoening, B. D. Schoenrock, E. Schopf, M. Schott, J. Schovancova, S. Schramm, M. Schreyer, N. Schuh, M. J. Schultens, H.-C. Schultz-Coulon, H. Schulz, M. Schumacher, B. A. Schumm, Ph. Schune, A. Schwartzman, T. A. Schwarz, Ph. Schwegler, H. Schweiger, Ph. Schwemling, R. Schwienhorst, J. Schwindling, T. Schwindt, G. Sciolla, F. Scuri, F. Scutti, J. Searcy, P. Seema, S. C. Seidel, A. Seiden, F. Seifert, J. M. Seixas, G. Sekhniaidze, K. Sekhon, S. J. Sekula, D. M. Seliverstov, N. Semprini-Cesari, C. Serfon, L. Serin, L. Serkin, M. Sessa, R. Seuster, H. Severini, T. Sfiligoj, F. Sforza, A. Sfyrla, E. Shabalina, N. W. Shaikh, L. Y. Shan, R. Shang, J. T. Shank, M. Shapiro, P. B. Shatalov, K. Shaw, S. M. Shaw, A. Shcherbakova, C. Y. Shehu, P. Sherwood, L. Shi, S. Shimizu, C. O. Shimmin, M. Shimojima, M. Shiyakova, A. Shmeleva, D. Shoaleh Saadi, M. J. Shochet, S. Shojaii, S. Shrestha, E. Shulga, M. A. Shupe, P. Sicho, A. M. Sickles, P. E. Sidebo, O. Sidiropoulou, D. Sidorov, A. Sidoti, F. Siegert, Dj. Sijacki, J. Silva, S. B. Silverstein, V. Simak, O. Simard, Lj. Simic, S. Simion, E. Simioni, B. Simmons, D. Simon, M. Simon, P. Sinervo, N. B. Sinev, M. Sioli, G. Siragusa, S. Yu. Sivoklokov, J. Sjölin, T. B. Sjursen, M. B. Skinner, H. P. Skottowe, P. Skubic, M. Slater, T. Slavicek, M. Slawinska, K. Sliwa, R. Slovak, V. Smakhtin, B. H. Smart, L. Smestad, J. Smiesko, S. Yu. Smirnov, Y. Smirnov, L. N. Smirnova, O. Smirnova, M. N. K. Smith, R. W. Smith, M. Smizanska, K. Smolek, A. A. Snesarev, S. Snyder, R. Sobie, F. Socher, A. Soffer, D. A. Soh, G. Sokhrannyi, C. A. Solans Sanchez, M. Solar, E. Yu. Soldatov, U. Soldevila, A. A. Solodkov, A. Soloshenko, O. V. Solovyanov, V. Solovyev, P. Sommer, H. Son, H. Y. Song, A. Sood, A. Sopczak, V. Sopko, V. Sorin, D. Sosa, C. L. Sotiropoulou, R. Soualah, A. M. Soukharev, D. South, B. C. Sowden, S. Spagnolo, M. Spalla, M. Spangenberg, F. Spanò, D. Sperlich, F. Spettel, R. Spighi, G. Spigo, L. A. Spiller, M. Spousta, R. D. St. Denis, A. Stabile, R. Stamen, S. Stamm, E. Stanecka, R. W. Stanek, C. Stanescu, M. Stanescu-Bellu, M. M. Stanitzki, S. Stapnes, E. A. Starchenko, G. H. Stark, J. Stark, P. Staroba, P. Starovoitov, S. Stärz, R. Staszewski, P. Steinberg, B. Stelzer, H. J. Stelzer, O. Stelzer-Chilton, H. Stenzel, G. A. Stewart, J. A. Stillings, M. C. Stockton, M. Stoebe, G. Stoicea, P. Stolte, S. Stonjek, A. R. Stradling, A. Straessner, M. E. Stramaglia, J. Strandberg, S. Strandberg, A. Strandlie, M. Strauss, P. Strizenec, R. Ströhmer, D. M. Strom, R. Stroynowski, A. Strubig, S. A. Stucci, B. Stugu, N. A. Styles, D. Su, J. Su, R. Subramaniam, S. Suchek, Y. Sugaya, M. Suk, V. V. Sulin, S. Sultansoy, T. Sumida, S. Sun, X. Sun, J. E. Sundermann, K. Suruliz, G. Susinno, M. R. Sutton, S. Suzuki, M. Svatos, M. Swiatlowski, I. Sykora, T. Sykora, D. Ta, C. Taccini, K. Tackmann, J. Taenzer, A. Taffard, R. Tafirout, N. Taiblum, H. Takai, R. Takashima, T. Takeshita, Y. Takubo, M. Talby, A. A. Talyshev, K. G. Tan, J. Tanaka, R. Tanaka, S. Tanaka, B. B. Tannenwald, S. Tapia Araya, S. Tapprogge, S. Tarem, G. F. Tartarelli, P. Tas, M. Tasevsky, T. Tashiro, E. Tassi, A. Tavares Delgado, Y. Tayalati, A. C. Taylor, G. N. Taylor, P. T. E. Taylor, W. Taylor, F. A. Teischinger, P. Teixeira-Dias, K. K. Temming, D. Temple, H. Ten Kate, P. K. Teng, J. J. Teoh, F. Tepel, S. Terada, K. Terashi, J. Terron, S. Terzo, M. Testa, R. J. Teuscher, T. Theveneaux-Pelzer, J. P. Thomas, J. Thomas-Wilsker, E. N. Thompson, P. D. Thompson, A. S. Thompson, L. A. Thomsen, E. Thomson, M. Thomson, M. J. Tibbetts, R. E. Ticse Torres, V. O. Tikhomirov, Yu. A. Tikhonov, S. Timoshenko, P. Tipton, S. Tisserant, K. Todome, T. Todorov, S. Todorova-Nova, J. Tojo, S. Tokár, K. Tokushuku, E. Tolley, L. Tomlinson, M. Tomoto, L. Tompkins, K. Toms, B. Tong, E. Torrence, H. Torres, E. Torró Pastor, J. Toth, F. Touchard, D. R. Tovey, T. Trefzger, A. Tricoli, I. M. Trigger, S. Trincaz-Duvoid, M. F. Tripiana, W. Trischuk, B. Trocmé, A. Trofymov, C. Troncon, M. Trottier-McDonald, M. Trovatelli, L. Truong, M. Trzebinski, A. Trzupek, J. C-L. Tseng, P. V. Tsiareshka, G. Tsipolitis, N. Tsirintanis, S. Tsiskaridze, V. Tsiskaridze, E. G. Tskhadadze, K. M. Tsui, I. I. Tsukerman, V. Tsulaia, S. Tsuno, D. Tsybychev, A. Tudorache, V. Tudorache, A. N. Tuna, S. A. Tupputi, S. Turchikhin, D. Turecek, D. Turgeman, R. Turra, A. J. Turvey, P. M. Tuts, M. Tyndel, G. Ucchielli, I. Ueda, R. Ueno, M. Ughetto, F. Ukegawa, G. Unal, A. Undrus, G. Unel, F. C. Ungaro, Y. Unno, C. Unverdorben, J. Urban, P. Urquijo, P. Urrejola, G. Usai, A. Usanova, L. Vacavant, V. Vacek, B. Vachon, C. Valderanis, E. Valdes Santurio, N. Valencic, S. Valentinetti, A. Valero, L. Valery, S. Valkar, S. Vallecorsa, J. A. Valls Ferrer, W. Van Den Wollenberg, P. C. Van Der Deijl, R. van der Geer, H. van der Graaf, N. van Eldik, P. van Gemmeren, J. Van Nieuwkoop, I. van Vulpen, M. C. van Woerden, M. Vanadia, W. Vandelli, R. Vanguri, A. Vaniachine, P. Vankov, G. Vardanyan, R. Vari, E. W. Varnes, T. Varol, D. Varouchas, A. Vartapetian, K. E. Varvell, J. G. Vasquez, F. Vazeille, T. Vazquez Schroeder, J. Veatch, L. M. Veloce, F. Veloso, S. Veneziano, A. Ventura, M. Venturi, N. Venturi, A. Venturini, V. Vercesi, M. Verducci, W. Verkerke, J. C. Vermeulen, A. Vest, M. C. Vetterli, O. Viazlo, I. Vichou, T. Vickey, O. E. Vickey Boeriu, G. H. A. Viehhauser, S. Viel, L. Vigani, R. Vigne, M. Villa, M. Villaplana Perez, E. Vilucchi, M. G. Vincter, V. B. Vinogradov, C. Vittori, I. Vivarelli, S. Vlachos, M. Vlasak, M. Vogel, P. Vokac, G. Volpi, M. Volpi, H. von der Schmitt, E. von Toerne, V. Vorobel, K. Vorobev, M. Vos, R. Voss, J. H. Vossebeld, N. Vranjes, M. Vranjes Milosavljevic, V. Vrba, M. Vreeswijk, R. Vuillermet, I. Vukotic, Z. Vykydal, P. Wagner, W. Wagner, H. Wahlberg, S. Wahrmund, J. Wakabayashi, J. Walder, R. Walker, W. Walkowiak, V. Wallangen, C. Wang, C. Wang, F. Wang, H. Wang, H. Wang, J. Wang, J. Wang, K. Wang, R. Wang, S. M. Wang, T. Wang, T. Wang, W. Wang, X. Wang, C. Wanotayaroj, A. Warburton, C. P. Ward, D. R. Wardrope, A. Washbrook, P. M. Watkins, A. T. Watson, M. F. Watson, G. Watts, S. Watts, B. M. Waugh, S. Webb, M. S. Weber, S. W. Weber, J. S. Webster, A. R. Weidberg, B. Weinert, J. Weingarten, C. Weiser, H. Weits, P. S. Wells, T. Wenaus, T. Wengler, S. Wenig, N. Wermes, M. Werner, M. D. Werner, P. Werner, M. Wessels, J. Wetter, K. Whalen, N. L. Whallon, A. M. Wharton, A. White, M. J. White, R. White, D. Whiteson, F. J. Wickens, W. Wiedenmann, M. Wielers, P. Wienemann, C. Wiglesworth, L. A. M. Wiik-Fuchs, A. Wildauer, F. Wilk, H. G. Wilkens, H. H. Williams, S. Williams, C. Willis, S. Willocq, J. A. Wilson, I. Wingerter-Seez, F. Winklmeier, O. J. Winston, B. T. Winter, M. Wittgen, J. Wittkowski, S. J. Wollstadt, M. W. Wolter, H. Wolters, B. K. Wosiek, J. Wotschack, M. J. Woudstra, K. W. Wozniak, M. Wu, M. Wu, S. L. Wu, X. Wu, Y. Wu, T. R. Wyatt, B. M. Wynne, S. Xella, D. Xu, L. Xu, B. Yabsley, S. Yacoob, R. Yakabe, D. Yamaguchi, Y. Yamaguchi, A. Yamamoto, S. Yamamoto, T. Yamanaka, K. Yamauchi, Y. Yamazaki, Z. Yan, H. Yang, H. Yang, Y. Yang, Z. Yang, W-M. Yao, Y. C. Yap, Y. Yasu, E. Yatsenko, K. H. Yau Wong, J. Ye, S. Ye, I. Yeletskikh, A. L. Yen, E. Yildirim, K. Yorita, R. Yoshida, K. Yoshihara, C. Young, C. J. S. Young, S. Youssef, D. R. Yu, J. Yu, J. M. Yu, J. Yu, L. Yuan, S. P. Y. Yuen, I. Yusuff, B. Zabinski, R. Zaidan, A. M. Zaitsev, N. Zakharchuk, J. Zalieckas, A. Zaman, S. Zambito, L. Zanello, D. Zanzi, C. Zeitnitz, M. Zeman, A. Zemla, J. C. Zeng, Q. Zeng, K. Zengel, O. Zenin, T. Ženiš, D. Zerwas, D. Zhang, F. Zhang, G. Zhang, H. Zhang, J. Zhang, L. Zhang, R. Zhang, R. Zhang, X. Zhang, Z. Zhang, X. Zhao, Y. Zhao, Z. Zhao, A. Zhemchugov, J. Zhong, B. Zhou, C. Zhou, L. Zhou, L. Zhou, M. Zhou, N. Zhou, C. G. Zhu, H. Zhu, J. Zhu, Y. Zhu, X. Zhuang, K. Zhukov, A. Zibell, D. Zieminska, N. I. Zimine, C. Zimmermann, S. Zimmermann, Z. Zinonos, M. Zinser, M. Ziolkowski, L. Živković, G. Zobernig, A. Zoccoli, M. zur Nedden, G. Zurzolo, L. Zwalinski, ATLAS Collaboration

**Affiliations:** 1 Department of Physics, University of Adelaide, Adelaide, SA Australia; 2 Physics Department, SUNY Albany, Albany, NY USA; 3 Department of Physics, University of Alberta, Edmonton, AB Canada; 4 Department of Physics, Ankara University, Ankara, Turkey; 5 Istanbul Aydin University, Istanbul, Turkey; 6 Division of Physics, TOBB University of Economics and Technology, Ankara, Turkey; 7 LAPP, CNRS/IN2P3 and Université Savoie Mont Blanc, Annecy-le-Vieux, France; 8 High Energy Physics Division, Argonne National Laboratory, Argonne, IL USA; 9 Department of Physics, University of Arizona, Tucson, AZ USA; 10 Department of Physics, The University of Texas at Arlington, Arlington, TX USA; 11 Physics Department, University of Athens, Athens, Greece; 12 Physics Department, National Technical University of Athens, Zografou, Greece; 13 Department of Physics, The University of Texas at Austin, Austin, TX United States of America; 14 Institute of Physics, Azerbaijan Academy of Sciences, Baku, Azerbaijan; 15 Institut de Física d’Altes Energies (IFAE), The Barcelona Institute of Science and Technology, Barcelona, Spain; 16 Institute of Physics, University of Belgrade, Belgrade, Serbia; 17 Vinca Institute of Nuclear Sciences, University of Belgrade, Belgrade, Serbia; 18 Department for Physics and Technology, University of Bergen, Bergen, Norway; 19 Physics Division, Lawrence Berkeley National Laboratory and University of California, Berkeley, CA USA; 20 Department of Physics, Humboldt University, Berlin, Germany; 21 Albert Einstein Center for Fundamental Physics and Laboratory for High Energy Physics, University of Bern, Bern, Switzerland; 22 School of Physics and Astronomy, University of Birmingham, Birmingham, UK; 23 Department of Physics, Bogazici University, Istanbul, Turkey; 24 Department of Physics Engineering, Gaziantep University, Gaziantep, Turkey; 25 Istanbul Bilgi University, Faculty of Engineering and Natural Sciences, Istanbul, Turkey; 26 Bahcesehir University, Faculty of Engineering and Natural Sciences, Istanbul, Turkey; 27 Centro de Investigaciones, Universidad Antonio Narino, Bogota, Colombia; 28 INFN Sezione di Bologna, Bologna, Italy; 29 Dipartimento di Fisica e Astronomia, Università di Bologna, Bologna, Italy; 30 Physikalisches Institut, University of Bonn, Bonn, Germany; 31 Department of Physics, Boston University, Boston, MA USA; 32 Department of Physics, Brandeis University, Waltham, MA USA; 33 Universidade Federal do Rio De Janeiro COPPE/EE/IF, Rio de Janeiro, Brazil; 34 Electrical Circuits Department, Federal University of Juiz de Fora (UFJF), Juiz de Fora, Brazil; 35 Federal University of Sao Joao del Rei (UFSJ), São João del Rei, Brazil; 36 Instituto de Fisica, Universidade de Sao Paulo, São Paulo, Brazil; 37 Physics Department, Brookhaven National Laboratory, Upton, NY USA; 38 Transilvania University of Brasov, Brasov, Romania; 39 National Institute of Physics and Nuclear Engineering, Bucharest, Romania; 40 Physics Department, National Institute for Research and Development of Isotopic and Molecular Technologies, Cluj Napoca, Romania; 41 University Politehnica Bucharest, Bucharest, Romania; 42 West University in Timisoara, Timisoara, Romania; 43 Departamento de Física, Universidad de Buenos Aires, Buenos Aires, Argentina; 44 Cavendish Laboratory, University of Cambridge, Cambridge, UK; 45 Department of Physics, Carleton University, Ottawa, ON Canada; 46 CERN, Geneva, Switzerland; 47 Enrico Fermi Institute, University of Chicago, Chicago, IL USA; 48 Departamento de Física, Pontificia Universidad Católica de Chile, Santiago, Chile; 49 Departamento de Física, Universidad Técnica Federico Santa María, Valparaiso, Chile; 50 Institute of High Energy Physics, Chinese Academy of Sciences, Beijing, China; 51 Department of Modern Physics, University of Science and Technology of China, Hefei, Anhui China; 52 Department of Physics, Nanjing University, Nanjing, Jiangsu China; 53 School of Physics, Shandong University, Jinan, Shandong China; 54 Department of Physics and Astronomy, Shanghai Key Laboratory for Particle Physics and Cosmology, Shanghai Jiao Tong University, also affiliated with PKU-CHEP, Shanghai, China; 55 Physics Department, Tsinghua University, Beijing, 100084 China; 56 Laboratoire de Physique Corpusculaire, Clermont Université and Université Blaise Pascal and CNRS/IN2P3, Clermont-Ferrand, France; 57 Nevis Laboratory, Columbia University, Irvington, NY USA; 58 Niels Bohr Institute, University of Copenhagen, Copenhagen, Denmark; 59 INFN Gruppo Collegato di Cosenza, Laboratori Nazionali di Frascati, Frascati, Italy; 60 Dipartimento di Fisica, Università della Calabria, Rende, Italy; 61 Faculty of Physics and Applied Computer Science, AGH University of Science and Technology, Kraków, Poland; 62 Marian Smoluchowski Institute of Physics, Jagiellonian University, Kraków, Poland; 63 Institute of Nuclear Physics, Polish Academy of Sciences, Kraków, Poland; 64 Physics Department, Southern Methodist University, Dallas, TX USA; 65 Physics Department, University of Texas at Dallas, Richardson, TX USA; 66 DESY, Hamburg, Zeuthen, Germany; 67 Institut für Experimentelle Physik IV, Technische Universität Dortmund, Dortmund, Germany; 68 Institut für Kern-und Teilchenphysik, Technische Universität Dresden, Dresden, Germany; 69 Department of Physics, Duke University, Durham, NC USA; 70 SUPA-School of Physics and Astronomy, University of Edinburgh, Edinburgh, UK; 71 INFN Laboratori Nazionali di Frascati, Frascati, Italy; 72 Fakultät für Mathematik und Physik, Albert-Ludwigs-Universität, Freiburg, Germany; 73 Section de Physique, Université de Genève, Geneva, Switzerland; 74 INFN Sezione di Genova, Genoa, Italy; 75 Dipartimento di Fisica, Università di Genova, Genoa, Italy; 76 E. Andronikashvili Institute of Physics, Iv. Javakhishvili Tbilisi State University, Tbilisi, Georgia; 77 High Energy Physics Institute, Tbilisi State University, Tbilisi, Georgia; 78 II Physikalisches Institut, Justus-Liebig-Universität Giessen, Giessen, Germany; 79 SUPA-School of Physics and Astronomy, University of Glasgow, Glasgow, UK; 80 II Physikalisches Institut, Georg-August-Universität, Göttingen, Germany; 81 Laboratoire de Physique Subatomique et de Cosmologie, Université Grenoble-Alpes, CNRS/IN2P3, Grenoble, France; 82 Department of Physics, Hampton University, Hampton, VA USA; 83 Laboratory for Particle Physics and Cosmology, Harvard University, Cambridge, MA USA; 84 Kirchhoff-Institut für Physik, Ruprecht-Karls-Universität Heidelberg, Heidelberg, Germany; 85 Physikalisches Institut, Ruprecht-Karls-Universität Heidelberg, Heidelberg, Germany; 86 ZITI Institut für technische Informatik, Ruprecht-Karls-Universität Heidelberg, Mannheim, Germany; 87 Faculty of Applied Information Science, Hiroshima Institute of Technology, Hiroshima, Japan; 88 Department of Physics, The Chinese University of Hong Kong, Shatin, N.T. Hong Kong; 89 Department of Physics, The University of Hong Kong, Hong Kong, China; 90 Department of Physics, The Hong Kong University of Science and Technology, Clear Water Bay, Kowloon, Hong Kong, China; 91 Department of Physics, Indiana University, Bloomington, IN USA; 92 Institut für Astro- und Teilchenphysik, Leopold-Franzens-Universität, Innsbruck, Austria; 93 University of Iowa, Iowa City, IA USA; 94 Department of Physics and Astronomy, Iowa State University, Ames, IA USA; 95 Joint Institute for Nuclear Research, JINR Dubna, Dubna, Russia; 96 KEK, High Energy Accelerator Research Organization, Tsukuba, Japan; 97 Graduate School of Science, Kobe University, Kobe, Japan; 98 Faculty of Science, Kyoto University, Kyoto, Japan; 99 Kyoto University of Education, Kyoto, Japan; 100 Department of Physics, Kyushu University, Fukuoka, Japan; 101 Instituto de Física La Plata, Universidad Nacional de La Plata and CONICET, La Plata, Argentina; 102 Physics Department, Lancaster University, Lancaster, UK; 103 INFN Sezione di Lecce, Lecce, Italy; 104 Dipartimento di Matematica e Fisica, Università del Salento, Lecce, Italy; 105 Oliver Lodge Laboratory, University of Liverpool, Liverpool, UK; 106 Department of Physics, Jožef Stefan Institute and University of Ljubljana, Ljubljana, Slovenia; 107 School of Physics and Astronomy, Queen Mary University of London, London, UK; 108 Department of Physics, Royal Holloway University of London, Surrey, UK; 109 Department of Physics and Astronomy, University College London, London, UK; 110 Louisiana Tech University, Ruston, LA USA; 111 Laboratoire de Physique Nucléaire et de Hautes Energies, UPMC and Université Paris-Diderot and CNRS/IN2P3, Paris, France; 112 Fysiska institutionen, Lunds universitet, Lund, Sweden; 113 Departamento de Fisica Teorica C-15, Universidad Autonoma de Madrid, Madrid, Spain; 114 Institut für Physik, Universität Mainz, Mainz, Germany; 115 School of Physics and Astronomy, University of Manchester, Manchester, UK; 116 CPPM, Aix-Marseille Université and CNRS/IN2P3, Marseille, France; 117 Department of Physics, University of Massachusetts, Amherst, MA USA; 118 Department of Physics, McGill University, Montreal, QC Canada; 119 School of Physics, University of Melbourne, Melbourne, VIC Australia; 120 Department of Physics, The University of Michigan, Ann Arbor, MI USA; 121 Department of Physics and Astronomy, Michigan State University, East Lansing, MI USA; 122 INFN Sezione di Milano, Milan, Italy; 123 Dipartimento di Fisica, Università di Milano, Milan, Italy; 124 B.I. Stepanov Institute of Physics, National Academy of Sciences of Belarus, Minsk, Republic of Belarus; 125 National Scientific and Educational Centre for Particle and High Energy Physics, Minsk, Republic of Belarus; 126 Group of Particle Physics, University of Montreal, Montreal, QC Canada; 127 P.N. Lebedev Physical Institute of the Russian Academy of Sciences, Moscow, Russia; 128 Institute for Theoretical and Experimental Physics (ITEP), Moscow, Russia; 129 National Research Nuclear University (MEPhI), Moscow, Russia; 130 D.V. Skobeltsyn Institute of Nuclear Physics, M.V. Lomonosov Moscow State University, Moscow, Russia; 131 Fakultät für Physik, Ludwig-Maximilians-Universität München, Munich, Germany; 132 Max-Planck-Institut für Physik (Werner-Heisenberg-Institut), Munich, Germany; 133 Nagasaki Institute of Applied Science, Nagasaki, Japan; 134 Graduate School of Science and Kobayashi-Maskawa Institute, Nagoya University, Nagoya, Japan; 135 INFN Sezione di Napoli, Naples, Italy; 136 Dipartimento di Fisica, Università di Napoli, Naples, Italy; 137 Department of Physics and Astronomy, University of New Mexico, Albuquerque, NM USA; 138 Institute for Mathematics, Astrophysics and Particle Physics, Radboud University Nijmegen/Nikhef, Nijmegen, The Netherlands; 139 Nikhef National Institute for Subatomic Physics and University of Amsterdam, Amsterdam, The Netherlands; 140 Department of Physics, Northern Illinois University, DeKalb, IL USA; 141 Budker Institute of Nuclear Physics, SB RAS, Novosibirsk, Russia; 142 Department of Physics, New York University, New York, NY USA; 143 Ohio State University, Columbus, OH USA; 144 Faculty of Science, Okayama University, Okayama, Japan; 145 Homer L. Dodge Department of Physics and Astronomy, University of Oklahoma, Norman, OK USA; 146 Department of Physics, Oklahoma State University, Stillwater, OK USA; 147 Palacký University, RCPTM, Olomouc, Czech Republic; 148 Center for High Energy Physics, University of Oregon, Eugene, OR USA; 149 LAL, Univ. Paris-Sud, CNRS/IN2P3, Université Paris Saclay, Orsay, France; 150 Graduate School of Science, Osaka University, Osaka, Japan; 151 Department of Physics, University of Oslo, Oslo, Norway; 152 Department of Physics, Oxford University, Oxford, UK; 153 INFN Sezione di Pavia, Pavia, Italy; 154 Dipartimento di Fisica, Università di Pavia, Pavia, Italy; 155 Department of Physics, University of Pennsylvania, Philadelphia, PA USA; 156 National Research Centre “Kurchatov Institute” B.P.Konstantinov Petersburg Nuclear Physics Institute, St. Petersburg, Russia; 157 INFN Sezione di Pisa, Pisa, Italy; 158 Dipartimento di Fisica E. Fermi, Università di Pisa, Pisa, Italy; 159 Department of Physics and Astronomy, University of Pittsburgh, Pittsburgh, PA USA; 160 Laboratório de Instrumentação e Física Experimental de Partículas -LIP, Lisbon, Portugal; 161 Faculdade de Ciências, Universidade de Lisboa, Lisbon, Portugal; 162 Department of Physics, University of Coimbra, Coimbra, Portugal; 163 Centro de Física Nuclear da Universidade de Lisboa, Lisbon, Portugal; 164 Departamento de Fisica, Universidade do Minho, Braga, Portugal; 165 Departamento de Fisica Teorica y del Cosmos and CAFPE, Universidad de Granada, Granada, Spain; 166 Dep Fisica and CEFITEC of Faculdade de Ciencias e Tecnologia, Universidade Nova de Lisboa, Caparica, Portugal; 167 Institute of Physics, Academy of Sciences of the Czech Republic, Prague, Czech Republic; 168 Czech Technical University in Prague, Prague, Czech Republic; 169 Faculty of Mathematics and Physics, Charles University in Prague, Prague, Czech Republic; 170 State Research Center Institute for High Energy Physics, Protvino, Russia; 171 Particle Physics Department, Rutherford Appleton Laboratory, Didcot NRC KI, UK; 172 INFN Sezione di Roma, Rome, Italy; 173 Dipartimento di Fisica, Sapienza Università di Roma, Rome, Italy; 174 INFN Sezione di Roma Tor Vergata, Rome, Italy; 175 Dipartimento di Fisica, Università di Roma Tor Vergata, Rome, Italy; 176 INFN Sezione di Roma Tre, Rome, Italy; 177 Dipartimento di Matematica e Fisica, Università Roma Tre, Rome, Italy; 178 Faculté des Sciences Ain Chock, Réseau Universitaire de Physique des Hautes Energies-Université Hassan II, Casablanca, Morocco; 179 Centre National de l’Energie des Sciences Techniques Nucleaires, Rabat, Morocco; 180 Faculté des Sciences Semlalia, Université Cadi Ayyad, LPHEA-Marrakech, Marrakech, Morocco; 181 Faculté des Sciences, Université Mohamed Premier and LPTPM, Oujda, Morocco; 182 Faculté des Sciences, Université Mohammed V, Rabat, Morocco; 183 DSM/IRFU (Institut de Recherches sur les Lois Fondamentales de l’Univers), CEA Saclay (Commissariat à l’Energie Atomique et aux Energies Alternatives), Gif-sur-Yvette, France; 184 Santa Cruz Institute for Particle Physics, University of California Santa Cruz, Santa Cruz, CA USA; 185 Department of Physics, University of Washington, Seattle, WA USA; 186 Department of Physics and Astronomy, University of Sheffield, Sheffield, UK; 187 Department of Physics, Shinshu University, Nagano, Japan; 188 Fachbereich Physik, Universität Siegen, Siegen, Germany; 189 Department of Physics, Simon Fraser University, Burnaby, BC Canada; 190 SLAC National Accelerator Laboratory, Stanford, CA USA; 191 Faculty of Mathematics, Physics and Informatics, Comenius University, Bratislava, Slovak Republic; 192 Department of Subnuclear Physics, Institute of Experimental Physics of the Slovak Academy of Sciences, Kosice, Slovak Republic; 193 Department of Physics, University of Cape Town, Cape Town, South Africa; 194 Department of Physics, University of Johannesburg, Johannesburg, South Africa; 195 School of Physics, University of the Witwatersrand, Johannesburg, South Africa; 196 Department of Physics, Stockholm University, Stockholm, Sweden; 197 The Oskar Klein Centre, Stockholm, Sweden; 198 Physics Department, Royal Institute of Technology, Stockholm, Sweden; 199 Departments of Physics and Astronomy and Chemistry, Stony Brook University, Stony Brook, NY USA; 200 Department of Physics and Astronomy, University of Sussex, Brighton, UK; 201 School of Physics, University of Sydney, Sydney, NSW Australia; 202 Institute of Physics, Academia Sinica, Taipei, Taiwan; 203 Department of Physics, Technion: Israel Institute of Technology, Haifa, Israel; 204 Raymond and Beverly Sackler School of Physics and Astronomy, Tel Aviv University, Tel Aviv, Israel; 205 Department of Physics, Aristotle University of Thessaloniki, Thessaloniki, Greece; 206 International Center for Elementary Particle Physics and Department of Physics, The University of Tokyo, Tokyo, Japan; 207 Graduate School of Science and Technology, Tokyo Metropolitan University, Tokyo, Japan; 208 Department of Physics, Tokyo Institute of Technology, Tokyo, Japan; 209 Department of Physics, University of Toronto, Toronto, ON Canada; 210 TRIUMF, Vancouver, BC Canada; 211 Department of Physics and Astronomy, York University, Toronto, ON Canada; 212 Faculty of Pure and Applied Sciences, and Center for Integrated Research in Fundamental Science and Engineering, University of Tsukuba, Tsukuba, Japan; 213 Department of Physics and Astronomy, Tufts University, Medford, MA USA; 214 Department of Physics and Astronomy, University of California Irvine, Irvine, CA USA; 215 INFN Gruppo Collegato di Udine, Sezione di Trieste, Udine, Italy; 216 ICTP, Trieste, Italy; 217 Dipartimento di Chimica Fisica e Ambiente, Università di Udine, Udine, Italy; 218 Department of Physics and Astronomy, University of Uppsala, Uppsala, Sweden; 219 Department of Physics, University of Illinois, Urbana, IL USA; 220 Instituto de Fisica Corpuscular (IFIC) and Departamento de Fisica Atomica, Molecular y Nuclear and Departamento de Ingeniería Electrónica and Instituto de Microelectrónica de Barcelona (IMB-CNM), University of Valencia and CSIC, Valencia, Spain; 221 Department of Physics, University of British Columbia, Vancouver, BC Canada; 222 Department of Physics and Astronomy, University of Victoria, Victoria, BC Canada; 223 Department of Physics, University of Warwick, Coventry, UK; 224 Waseda University, Tokyo, Japan; 225 Department of Particle Physics, The Weizmann Institute of Science, Rehovot, Israel; 226 Department of Physics, University of Wisconsin, Madison, WI USA; 227 Fakultät für Physik und Astronomie, Julius-Maximilians-Universität, Würzburg, Germany; 228 Fakultät für Mathematik und Naturwissenschaften, Fachgruppe Physik, Bergische Universität Wuppertal, Wuppertal, Germany; 229 Department of Physics, Yale University, New Haven, CT USA; 230 Yerevan Physics Institute, Yerevan, Armenia; 231 Centre de Calcul de l’Institut National de Physique Nucléaire et de Physique des Particules (IN2P3), Villeurbanne, France; 232 CERN, 1211 Geneva 23, Switzerland

## Abstract

The dijet production cross section for jets containing a *b*-hadron (*b*-jets) has been measured in proton–proton collisions with a centre-of-mass energy of $\sqrt{s} = 7$ TeV, using the ATLAS detector at the LHC. The data used correspond to an integrated luminosity of $\mathrm 4.2\,\text {fb}^{-1}$. The cross section is measured for events with two identified *b*-jets with a transverse momentum $p_{\text {T}} > 20$ GeV and a minimum separation in the $\eta$–$\phi$ plane of $\Delta R = 0.4$. At least one of the jets in the event is required to have $p_{\text {T}} > 270$ GeV. The cross section is measured differentially as a function of dijet invariant mass, dijet transverse momentum, boost of the dijet system, and the rapidity difference, azimuthal angle and angular distance between the *b*-jets. The results are compared to different predictions of leading order and next-to-leading order perturbative quantum chromodynamics matrix elements supplemented with models for parton-showers and hadronization.

## Introduction

The measurement of jets containing a *b*-hadron (*b*-jets) produced in proton–proton collisions at the Large Hadron Collider (LHC) provides an important test of perturbative quantum chromodynamics (pQCD). Calculations of the *b*-quark production cross section have been performed at next-to-leading order of $\alpha _\mathrm{s}$ (NLO) in pQCD [1–4]. These calculations can be combined with different parton-shower and hadronisation models to generate simulated events which can be compared to data.

Cross sections for the production of a $b\bar{b}$ pair have been measured previously at the Tevatron [5–8], and at the LHC by the ATLAS [9, 10] and CMS [11] collaborations. These measurements agree with NLO predictions for well-separated *b*-jets, although *b*-jets with large transverse momenta in the central regions are not well described by simulations [9]. The results in Ref. [10] also agree with the NLO predictions; though small deviations are present at large transverse momenta in events with a *b*-jet and light-flavour jet (jet generated by a light quark). The CMS measurement found that in the phase-space region of small angular separation between the *b*-jets, there are substantial differences between data and NLO predictions, and among the NLO predictions themselves.

**Fig. 1:**
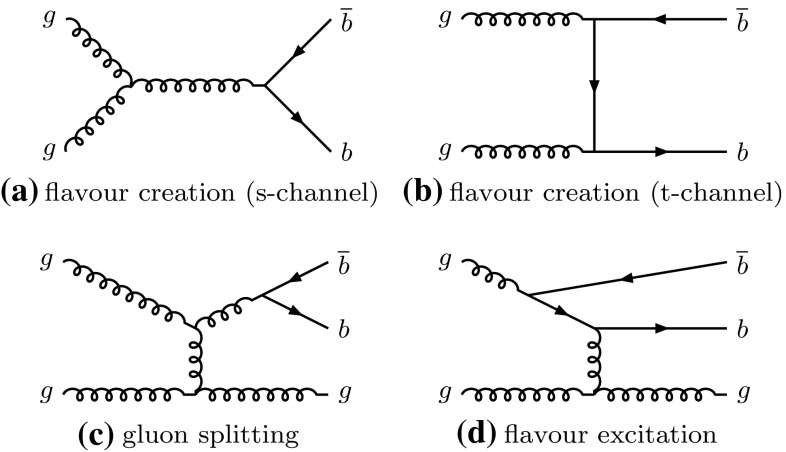
Lowest-order Feynman diagrams for $b\bar{b}$ production

The lowest-order Feynman diagrams for $b\bar{b}$ production are shown in Fig. 1. They define different production mechanisms which are useful in understanding the behaviour of the $b\bar{b}$ system. In *flavour creation* (FCR) both *b*-jets originate from the hard scatter: these jets tend to be the hardest in the event and are predicted to have an approximate back-to-back configuration in the transverse plane. The *gluon splitting* (GSP) production mechanism creates a pair of *b*-jets that are expected to have a small angular separation. The topology of *flavour excitation* (FEX) is less distinctive, but it tends to contain an additional parton, which reduces the angular separation between the *b*-jets. The requirement of a minimum transverse momentum ($p_{\text {T}}$) of 270 GeV for the leading jet applied in this analysis enhances the three-jet production mechanisms relative to the flavour creation mechanism, in comparison to the analyses of Refs. [9–11].

Different regions of the $b\bar{b}$ phase space are probed via the six differential cross sections presented in this article. For large values of the dijet invariant-mass, $m_{bb}$, the flavour creation mechanism is expected to dominate, leading to final states with well-separated hard jets. Events produced via gluon splitting or flavour excitation are concentrated at small $m_{bb}$. The opposite is expected for the $p_{\text {T}}$ 1 of the dijet system, $p_{{\mathrm T},bb}$, where the higher-$p_{{\mathrm T},bb}$ regions are dominated by gluon-splitting production, and only the lower values of $p_{{\mathrm T},bb}$ have significant contributions from events produced via flavour creation. The azimuthal angle between two *b*-jets, $\Delta \phi$, separates the different production mechanisms more evenly. The angular distance between the two *b*-jets, $\Delta R = \sqrt{(\Delta \phi )^2 + (\Delta \eta )^2}$, is a variable often used in analyses reconstructing heavy objects decaying into two *b*-jets. The other two observables are the rapidity difference between the two *b*-jets, $y^{*} = \frac{1}{2}|y_1 - y_2|$, where $y_{i}$ is the rapidity of *b*-jet *i*, and the boost of the dijet system, $y_B = \frac{1}{2}|y_1+y_2|$. The latter is related to the momentum of the initial-state partons involved in the hard scatter and it is therefore sensitive to the parton distribution functions (PDFs).

The measurement of the $b\bar{b}$ dijet differential cross sections is performed with the ATLAS detector, using proton–proton (*pp*) collisions at a centre-of-mass energy of $\sqrt{s} = 7$ TeV. The data were recorded in 2011 and correspond to an integrated luminosity of $\mathrm 4.2\, \text {fb}^{-1}$. The differential cross sections are defined as

1 $$\begin{aligned} \frac{{\mathrm d}\sigma \left( pp \rightarrow b\bar{b} + X\right) }{{\mathrm d}\mathcal {O}} = \frac{N_{\mathrm {\text {tag}}}f_{bb}\ \mathcal {U}}{\varepsilon \mathcal {L}\Delta \mathcal {O} }, \end{aligned}$$

with $\mathcal {O}$ the dijet observable under investigation, $N_{\mathrm {tag}}$ the number of *b*-tagged jet pairs, $f_{bb}$ the purity of the selected sample, $\varepsilon$ the selection efficiency, $\mathcal {L}$ the integrated luminosity and $\mathcal {U}$ the correction of the measured distribution for detector effects, such as the jet energy resolution. The measurement ranges for the different variables are listed in Table 1.

**Table 1:** Ranges of the variables of the measured differential cross sections

Variable	Range
$m_{bb}$	50–1000 GeV
$p_{{\mathrm T},bb}$	0–400 GeV
$\Delta \phi$	0–$\pi$
$\Delta R$	0.4–4.0
$y_B$	0–2.5
$y^*$	0–1.7

## ATLAS detector

The ATLAS detector [12] consists of an inner tracking system, immersed in a 2 T axial magnetic field, surrounded by electromagnetic calorimeters, hadronic calorimeters and a muon spectrometer.

The inner detector has full coverage in $\phi$ and covers the pseudorapidity range $|\eta | < 2.5$. The inner detector consists of silicon pixel and microstrip detectors, surrounded by a transition radiation tracker (up to $|\eta | = 2.0$). The electromagnetic calorimeter is a lead–liquid argon sampling calorimeter covering $|\eta | < 3.2$. Hadron calorimetry in the central pseudorapidity region ($|\eta | < 1.7$) is provided by a scintillator-tile calorimeter using steel as the absorber material. The hadronic end-cap calorimeter uses liquid argon with copper absorber plates and extends up to $|\eta | = 3.2$. Additional forward calorimeters extend the coverage to $|\eta | < 4.9$. The outer region of the detector is formed by a muon spectrometer that uses a toroidal magnetic field with a bending power of 1.5–5.5 Tm in the barrel and 1.0–7.5 Tm in the end-caps. The muon spectrometer provides trigger information for muons up to $|\eta | = 2.4$ and momentum measurements in the bending plane up to $|\eta | = 2.7$.

The trigger system uses three consecutive levels to record a selection of interesting events. The level-1 trigger (L1) is based on custom-built hardware that processes the data with a fixed latency of 2.5 ${\mathrm \mu }$s. The second level and the event filter, collectively referred to as the high-level trigger (HLT), are software-based triggers.

The jet triggers at L1 use information about the energy deposits in the electromagnetic and hadronic calorimeters using trigger towers with a granularity of $\Delta \phi \times \Delta \eta = 0.1 \times 0.1$. Jet identification is based on the transverse energy deposited in a sliding window of $4\times 4$ or $8\times 8$ trigger towers. The HLT further refines the selection, making use of finer-granularity detector information and using reconstruction software close to that used by physics analyses. Due to the high rate of jet production, only a predetermined fraction of events that pass the jet triggers are recorded. The factor by which the number of events that pass a trigger is reduced is known as the *prescale*.

## Simulated dataset

To investigate efficiencies and model the data, simulated dijet events produced by the Pythia 6.4  [13] Monte Carlo (MC) event generator are used. Pythia 6.4 implements matrix elements at leading order (LO) in $\alpha _\mathrm{s}$ for 2$\rightarrow$2 processes, a $p_{\text {T}}$-ordered parton shower with leading-logarithm accuracy and multi-parton interactions to simulate the underlying event. The hadronisation is described using the Lund string model [14]. Events are generated with the MRST LO** [15] PDFs and a set of parameters tuned to ATLAS data, AUET2B-LO** [16]. At this stage, all generated particles with a lifetime greater than 30 ps are collectively referred to as the *particle-level* event. *Detector-level* events are produced by passing the particle-level events through a full simulation [17] of the ATLAS detector based on GEANT4 [18]. The effect of multiple *pp* interactions in the same or nearby bunch crossings (pile-up) is included in all MC simulations. Events in MC simulation are reweighted, in order to match the distribution of the number of multiple *pp* interaction distributions to that observed in the data. During the 2011 data-taking period, the number of *pp* collisions per bunch crossing varied between 0.5 and 24 [19]. The resulting simulated events are digitised to model the detector responses, and then reconstructed using the same software as for data processing.

## Jet selection

Jets are reconstructed from energy clusters in the calorimeter using the anti-$k_t$  [20, 21] algorithm as implemented in the FastJet package [22], with jet radius parameter $R=0.4$. Jet energy is corrected to the hadronic energy scale [23], which on average adjusts the reconstructed jet energy to the true energy. The reconstructed jets are subjected to calorimeter-based quality selections [24]. Jet candidates coming from background processes, namely: cosmic-ray showers, LHC beam conditions and hardware problems, are rejected as described in Ref. [25]. Central jets with $|\eta |<2.5$ originating from pile-up are rejected by a track-based selection [26].

### *b*-Jet selection

The flavour of a jet at particle level is defined according to the hadrons contained in the jet. If the jet contains at least one *b*-hadron with $p_{\text {T}} > 5$ GeV and $\Delta R$ with respect to the jet axis of less than 0.3, then it is considered as a *b*-jet. If no *b*-hadron is present, but a *c*-hadron that meets the same criteria is found, then the jet is considered as a *c*-jet. All other jets are considered as light-flavour jets.

At detector level, the relatively long lifetime of *b*-hadrons is used to select an event sample enriched in *b*-jet pairs. To identify *b*-jets, a combination of the JetFitter and IP3D algorithms [27] is used. The JetFitter algorithm aims at reconstructing the decay vertex of the *b*-hadron and the subsequently produced *c*-hadron, assuming that both vertices lie on the same line from the primary vertex,^2^ corresponding to the flight direction of the *b*-hadron. The IP3D algorithm is a track-based algorithm using the signed longitudinal and transverse impact parameter significances of the tracks matched to the jet (where the impact parameter is defined as the distance of the track from the vertex at the point of closest approach). The variables describing the impact parameters and the reconstructed decay chain are combined by a neural network trained using MC simulation samples. This combination assigns a set of probabilities $(p_b, p_c, p_l)$ to every jet, corresponding to the probability of the jet being a *b*-jet, *c*-jet or light-flavour jet, respectively. A jet is considered to be *b*-tagged when $\log _{10}\left( p_b/p_l\right) > 0.35$, a choice that results in a *b*-tagging efficiency of $\varepsilon _b \sim 70\%$ in simulated $t\bar{t}$ events (corresponding to a *c*-jet rejection factor of 5 and light-flavour jet rejection factor of 125).

A scale factor is applied to the efficiency obtained from simulation to account for the data-MC difference. Two methods are employed to select these *b*-jet samples: the first uses an independent *b*-tagging algorithm that selects jets containing a muon from a semileptonic *b*-hadron decay [28]; the other selects *b*-jets from $t\bar{t}$ decays [29]. The differences between data and simulation observed in these control samples are used to derive a series of $p_{\text {T}}$- and $\eta$-dependent scale factors, which are then applied to each jet in simulation.

Table 2 contains all the fiducial phase-space definitions for particle-level and detector-level objects used in this analysis.

**Table 2:** Fiducial phase space of the measurement. The definition and the selection requirements for particle-level and detector-level jets are given. The particle-level jets are constructed using all particles, including muons and neutrinos, as input (see Sect. 6). The $\log _{10}\left( p_b/p_l\right)$ criterion corresponds to the ratio of the probabilities of the jet being a *b*-jet or light-flavour jet

Definition	Particle-level jets	Detector-level jets
Jet identification	anti-$k_t$ with $R=0.4$	anti-$k_t$ with $R=0.4$
	Include muons and neutrinos	
*b*-Jets definition	*b*-Hadron with $p_{\text {T}} > 5$ GeV	$\log _{10}\left( p_b/p_l\right) > 0.35$
	$\Delta$R (jet;*b*-hadron)<0.3	
Event selection		
Leading jet	$p_{\text {T}} > 270$ GeV and $|\eta | < 3.2$	
2 *b*-Jets selection	$p_{\text {T}} > 20$ GeV and $|\eta | < 2.5$	
	2 *b*-jets separated by $\Delta R ~>~0.4$	

## Event selection

Events are selected using two calorimeter-based single-jet triggers with a $p_{\text {T}}$-thresholds of 180 and 240 GeVand $|\eta | < 3.2$. These thresholds are used to define two ranges for the transverse momentum of the leading jet where the trigger efficiency is close to 100%: $270< p_{\text {T}} < 355$ GeV and $p_{\text {T}} > 355$ GeV, respectively. A prescale factor of 3.5 was applied to the 180  GeV threshold trigger; no prescale factor was applied to the 240  GeV threshold trigger.

Quality requirements are applied to ensure that the selected events are well measured. In addition to selecting only data from periods in which all sub-detectors were operating nominally, a veto is applied to reject specific events in which the calorimeters were suffering from noisy or inactive regions.

The leading jet is not required to be identified as a *b*-jet, but the selected events must have at least two *b*-tagged jets with $p_{\text {T}} > 20$ GeV and $|\eta | < 2.5$, and the two highest-$p_{\text {T}}$ *b*-tagged jets within the $|\eta |$ requirement are taken as the dijet pair. To avoid jets with significant overlap, the two *b*-tagged jets in the pair are also required to be separated by $\Delta R ~>~0.4$.

**Fig. 2:**
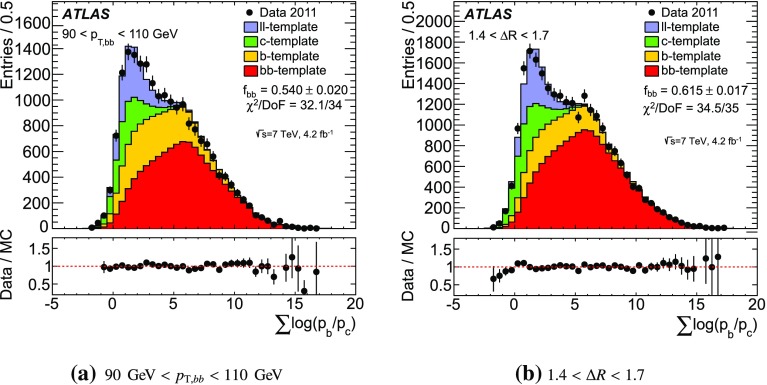
Examples of template fits in a $p_{{\mathrm T},bb}$ and $\Delta R$ bin. The uncertainty shown on the $b\bar{b}$ fraction is the statistical uncertainty of the fit parameter. Systematic uncertainties are not shown

### Purity

While the requirement of *b*-tagged jets provides an event sample enriched in $b\bar{b}$ pairs, there is still a non-negligible contamination from *c*-jets and light-flavour jets. The fraction of true *b*-jet pairs in the sample of *b*-tagged jet pairs, referred to as the purity of the sample, is determined by performing a template fit to the combined IP3D and JetFitter probability distributions. This fit is performed independently in each bin of the cross-section measurement. To obtain optimal separation between *b*- and *c*-jets, the fit variable is constructed as $\sum \log _{10}\left( p_b/p_c\right)$, where the sum is taken over both of the *b*-tagged jets.

The fit uses a maximum-likelihood method to determine the relative contributions of four templates that best describe the flavour content of the $b\bar{b}$ pair in data. These templates are defined as:

- *bb*-template: $$(f_1, f_2)$$ = (*b*, *b*),
- *b*-template: $$(f_1, f_2)$$ = (*b*, *c*), (*c*, *b*), (*b*, *l*) or (*l*, *b*),
- *c*-template: $$(f_1, f_2)$$ = (*c*, *c*), (*c*, *l*) or (*l*, *c*),
- *ll*-template: $$(f_1, f_2)$$ = (*l*, *l*),

where $f_1$ and $f_2$ indicate the flavour of the leading and sub-leading jet, respectively. The fraction of $b\bar{b}$ events in the *b*-tagged sample is determined by the relative contribution of the *bb*-template.

The dijet templates are obtained from single-jet templates in MC simulation using a convolution technique in every bin of the investigated variables. This allows the creation of smooth, finely binned templates even for bins with a small number of dijet pairs. As the *b*-hadron decay is a process internal to the jet, the *b*-tagger probabilities for a given jet do not depend significantly on the properties of the dijet system. The shape of the fit variable distribution can be parameterised as a function of the $p_{\text {T}}$ and flavour of the single jets in simulation. The contribution of each $(p_{\mathrm T1}, p_{\mathrm T2}, f_1, f_2)$ combination within a cross-section bin is then determined by convolving the $\log _{10}\left( p_b/p_c\right)$ distributions for $(p_{\mathrm T1}, f_1)$ and $(p_{\mathrm T2}, f_2)$. Figure 2 shows examples of the fits for two bins of the variables $p_{{\mathrm T},bb}$ and $\Delta R$.

To verify the validity of the procedure, a closure test is performed by comparing the templates obtained via the convolution to those obtained from the bi-dimensional $p_{\text {T}}$ distribution. Good agreement is observed with the generated templates in all kinematic regions.

## Unfolding

The correction of the measured distribution for detector effects and inefficiencies is done via an unfolding procedure that uses the iterative dynamically stabilised (IDS) method [30]. At particle level, jets are constructed using all particles, including muons and neutrinos, as input. Particle-level jets are required to pass the same kinematic selections as jets reconstructed in the calorimeter. The detector effects are corrected by using an unfolding matrix, which maps the event migrations in a binned distribution from detector level to particle level. The data are unfolded using the unfolding matrix and then compared to the predicted particle-level distribution. The iterative part of the unfolding allows the matrix to be modified to account for mismodelling of the MC simulation. Any statistically significant differences between the data and simulation are assumed to originate from processes not included in the simulation and are added into the unfolding matrix. The data are then unfolded using the modified unfolding matrix and the process is repeated until no element in the unfolding matrix is modified by more than one percent. To cross-check the unfolding results, the unfolding is done with a bin-by-bin method and compared. The bin-by-bin method takes the ratio of the detector-level jet and particle-level jet distributions, combining all the necessary corrections into a single factor. It treats each bin as an independent measurement, behaving as if events appear or disappear within the bin rather than moving to another. The ratio of IDS to bin-by-bin results is about 2%, except for the mass and the $p_{\text {T}}$, where differences up to 10% were observed.

The unfolding matrix is derived from simulated Pythia 6.4 dijet events, and is defined for events that have both the particle-level and the detector-level jet pairs within the fiducial acceptance of the analysis. Fiducial and efficiency correction factors are applied to the data before and after the unfolding, respectively. The fiducial correction, applied before the unfolding, accounts for the effects that cause a detector-level jet pair not to be matched to a particle-level jet pair. The primary reason for the mismatch is that one of the particle-level jets has a $p_{\text {T}}$ below the event selection threshold and so is rejected by the analysis. The efficiency corrections, applied after the unfolding, correct primarily for particle-level jets which meet the event selection criteria but are measured outside of the fiducial range at detector level.

## Systematic uncertainties 

The systematic uncertainties on the measured cross sections are evaluated varying the relevant quantities by one standard deviation, and applying the unfolding procedure; the differences with respect to the standard procedure are taken as the uncertainties. The total systematic uncertainties are obtained by adding the components in quadrature.

The dominant systematic uncertainties in this measurement result from the *b*-tagging and the jet energy scale calibrations. Table 3 provides an overview of the systematic uncertainties. Their magnitude depends on the variable used in the differential cross section measurement, and the minimum and maximum uncertainties are reported. Each systematic uncertainty is propagated through the entire analysis, including the unfolding procedure.

**Table 3:** Summary of the dominant sources of systematic uncertainties and their relative effect on the cross section

Source	Cross section relative uncertainty
*b*-Tagging efficiency	10–30%
*b*-Jet template fit	3–8%
Jet energy scale	10–20%
Jet energy resolution	2–8%
Jet angular resolution	1–5%
Unfolding	5–10%
Luminosity	1.8%

The *b*-tagging efficiency and light-flavour-jet rejection rates in MC simulation are calibrated by applying $p_{\text {T}}$- and $\eta$-dependent scale factors to the simulated jets [28, 29]. These scale factors are derived using various data-driven techniques, as discussed in Sect. 4.1. The uncertainty from this calibration is evaluated by varying the scale factors for each jet flavour by one standard deviation. The effect of this uncertainty ranges from 10 to 20% in most bins, and reaches 30% for low and high values of $m_{bb}$.

While the *b*-tagging calibration uncertainty takes into account differences between data and MC simulation in the *b*-tagging efficiency, this does not necessarily account for differences in the shape of the *b*-tagger probability distributions. The *b*-tagging algorithm used for this analysis makes use of tracks to identify *b*-jets. To account for any effects due to track mismodelling, all the template fits are re-evaluated after the simulated events are reweighted to match the small difference in *b*-jet track multiplicity observed in data and the difference is taken as the uncertainty. The resulting uncertainty in the cross section amounts to about 1%. In addition, a control sample of *b*-jets is selected using a tag-and-probe method. The data-to-MC ratio of the $\log _{10}\left( p_b/p_c\right)$ for the probe jets is fitted with a first-order polynomial. The template fits are then redone after the MC simulation is reweighted to match the difference in $\log _{10}\left( p_b/p_c\right)$ seen in data. The resulting uncertainty is in the range 3$-8\%$.

The systematic uncertainty resulting from the calibration of the jet energy scale [23] is typically around 10%, but reaches 20% for jets with a small angular separation. Smaller contributions to the jet uncertainties result from mismodelling of the jet energy resolution [31] and the resolution of the jet direction. The uncertainty of the jet energy resolution is estimated by performing an additional Gaussian smearing of the jets by one standard deviation, resulting in a 2–8% uncertainty. The uncertainty due to the jet angular resolution is estimated by comparing the angular resolution of the nominal sample with that of samples for which the material description and *b*-jet fragmentation are varied [32, 33]; this uncertainty is in the range 1–5%.

The unfolding uncertainty is evaluated by reweighting the MC simulation that is used to derive the unfolding matrix to reproduce the cross section measured in data. Using the reweighted unfolding matrix results in a 5–10% change in cross section, which is assigned as a systematic uncertainty. Finally, the systematic uncertainty of the luminosity, which is fully correlated among all bins, is 1.8% [19]. All other sources of correlations are assumed to be negligible.

## Theoretical predictions

The results are compared to the NLO MC generators Powheg, r2299, [34–37] and MC@NLO  4.01 [38, 39]. Both NLO generators use the CT10 [40] PDFs and a *b*-quark mass of 4.95 GeV. Events generated with Powheg are passed through the Pythia 6.4 parton shower and MC@NLO events are showered with Herwig 6.520 [41]. Herwig 6 uses an angular ordering parton shower with a cluster hadronisation model and employs the MRST LO** PDFs and the set of tuned parameters AUET2-LO** [42]. While Powheg and MC@NLO are both formally accurate to NLO, their treatment of higher-order terms differs. The data are also compared to the LO predictions provided by the Sherpa  1.43 [43] and Pythia 6.4 MC generators. Sherpa is capable of generating multiple partons in its matrix elements, and was also used to generate $b\bar{b}$ using a LO 2$\rightarrow$3 matrix elements for this prediction. As Pythia 6.4 is a LO generator, it is not expected to provide an accurate normalisation. The Pythia 6.4 distributions are normalised to the integrated cross section measured in data by applying a factor of 0.61. Sherpa is found to produce the correct cross-section normalisation. Powheg +Pythia 6.4 is chosen as the baseline to examine the theoretical uncertainties. The largest theoretical uncertainties derive from the PDF uncertainties and uncertainties due to missing higher orders. By varying the renormalisation scale, $\mu _\mathrm{R}$, and the factorisation scale, $\mu _\mathrm{F}$, which are set to the same value in Powheg, an estimate of the effects of the missing higher-order terms can be made. To evaluate the uncertainty, the scales are varied independently from one half to twice the central value, and the cross-section variations are added in quadrature. The effect of the scale uncertainties on the NLO prediction ranges from 20 to 50%, and dominates the theoretical uncertainty. The uncertainties due to the choice of PDFs are estimated from the 52 eigenvectors of the CT10 PDF set evaluated at 68% confidence level, and are in the range 5–10% for the variables investigated. Other cross-checks were performed, such as a study of the effect due to the *b*-quark mass uncertainty and of the scale matching between Powheg and the parton shower. All of these have a negligible effect. The total theoretical uncertainty is obtained by adding the scale and PDF uncertainties in quadrature.

## Results and discussion

**Fig. 3:**
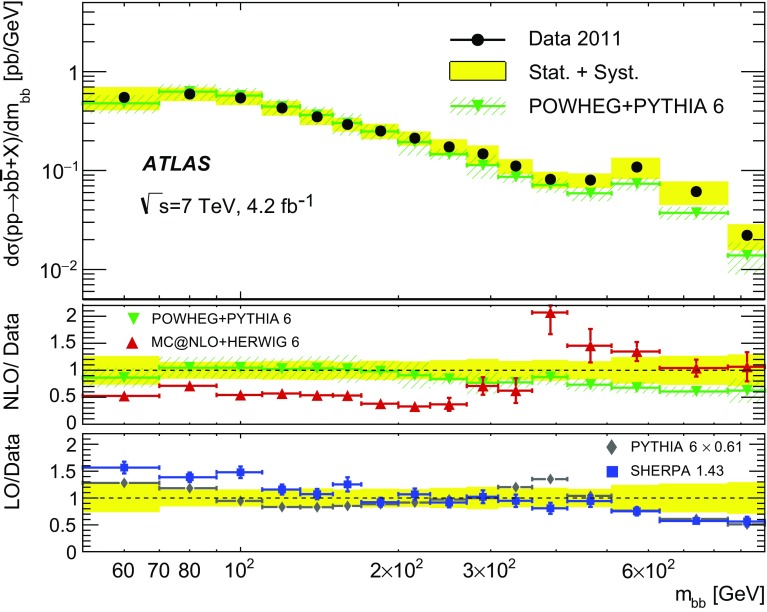
*Top panel* the differential cross section for $b\bar{b}$ production as a function of dijet invariant mass, $m_{bb}$, compared to the theoretical predictions obtained using Powheg. Theoretical uncertainties obtained by using Powheg are also shown. *Middle panel* ratio of the NLO predictions to the measured cross section. *Bottom panel* ratio of the LO predictions to the measured cross section. For the predictions from MC@NLO, Sherpa and Pythia 6.4 only the statistical uncertainties are shown. For both *Middle* and *Bottom panels* the *yellow band* represents the combined statistical and systematic experimental errors for the data. Theoretical uncertainties on the POWHEG prediction are also shown

**Fig. 4:**
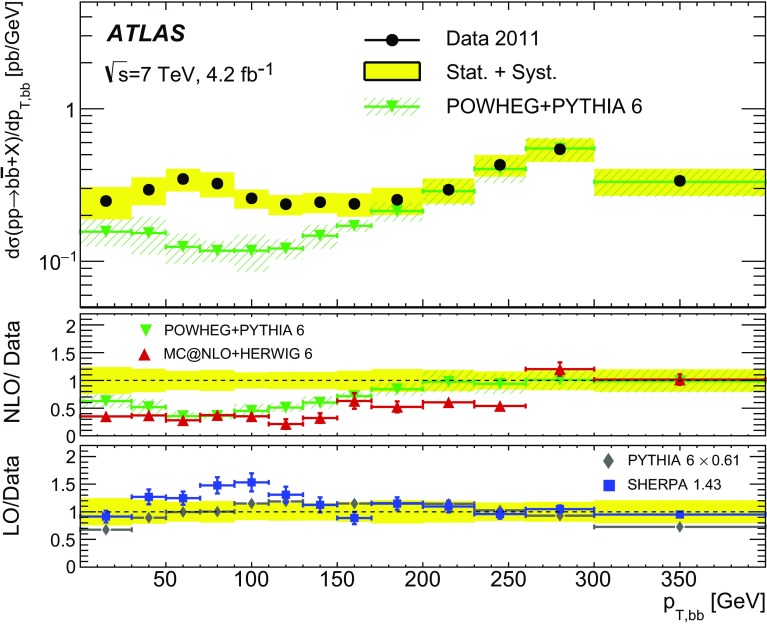
Differential cross section for $b\bar{b}$ production as a function of the transverse momentum of the dijet system, $p_{{\mathrm T},bb}$. The figure layout is as in Fig. 3

**Fig. 5:**
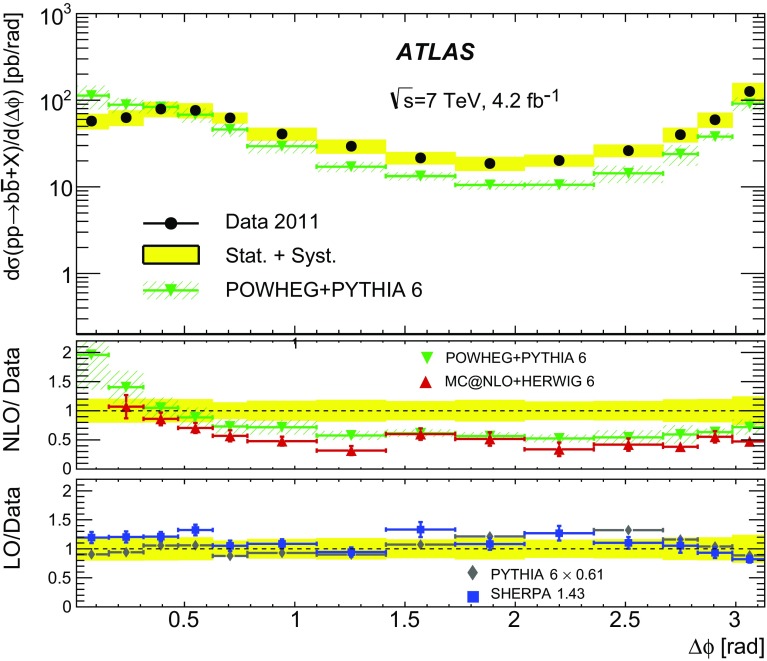
Differential cross section for $b\bar{b}$ production as a function of the azimuthal angle between the two jets, $\Delta \phi$. The point corresponding to the first bin of the MC@NLO to data ratio is about 3.5 and does not appear in the plot. The figure layout is as in Fig. 3

**Fig. 6:**
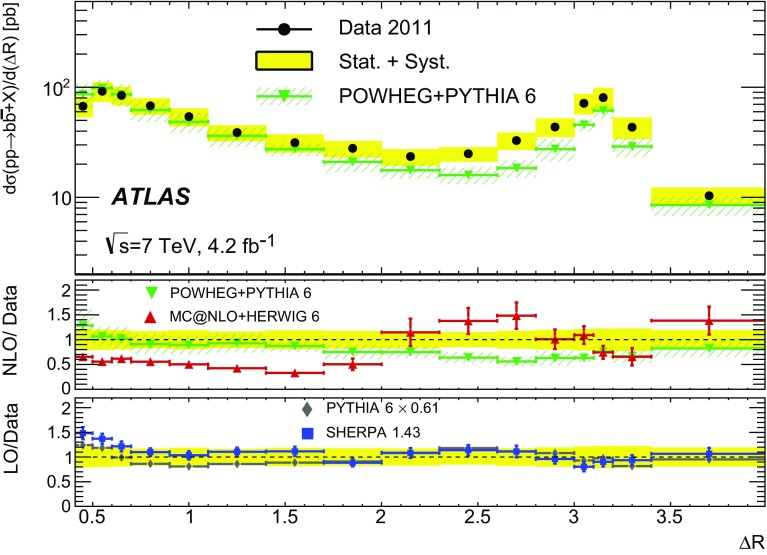
Differential cross section for $b\bar{b}$ production as a function of the angular distance between the two jets, $\Delta R = \sqrt{(\Delta \phi )^2 + (\Delta \eta )^2}$. The figure layout is as in Fig. 3

**Fig. 7:**
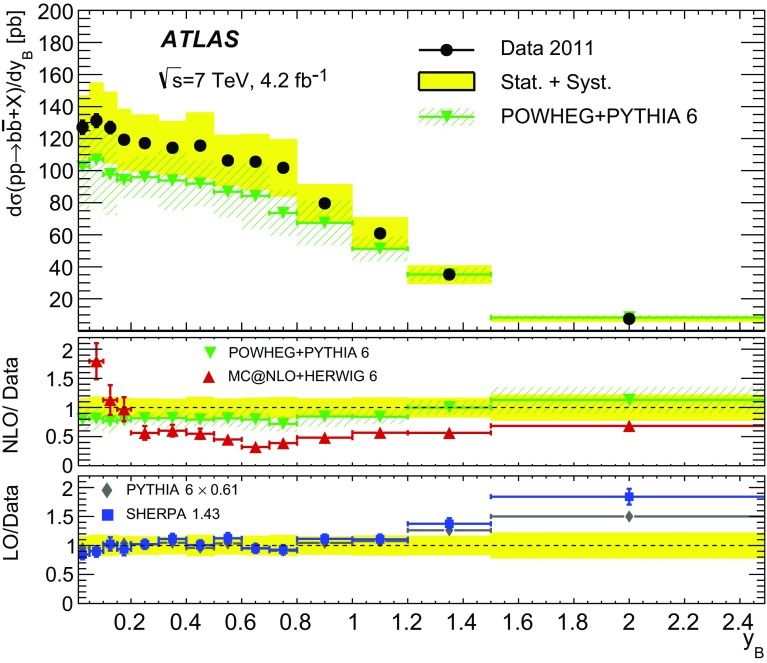
Differential cross section for $b\bar{b}$ production as a function of the boost of the dijet system, $y_B = \frac{1}{2}\left| y_1+y_2\right|$. The figure layout is as in Fig. 3

**Fig. 8:**
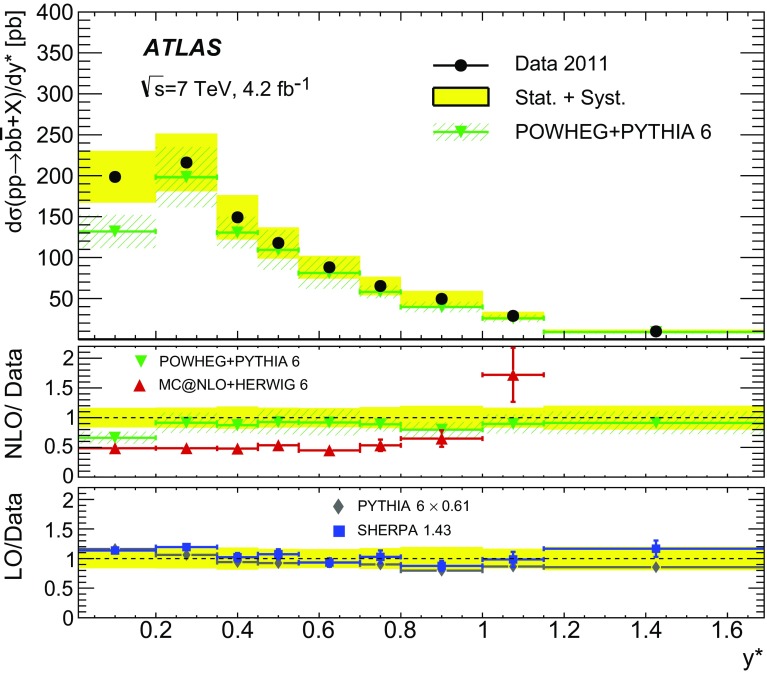
Differential cross section for $b\bar{b}$ production as a function of $y^{*} = \frac{1}{2}\left| y_1-y_2\right|$. The figure layout is as in Fig. 3

The differential cross section for $b\bar{b}$ production is shown as a function of the six observables in Figs. 3, 4, 5, 6, 7, 8. The top panel of each figure shows the data points as black dots, with the total experimental uncertainties as yellow boxes, together with the prediction and theoretical uncertainties obtained by using Powheg. The middle and lower panels report the ratio of theoretical predictions to data. For the predictions from MC@NLO, Sherpa and Pythia 6.4, only the statistical uncertainties are shown. Because of the normalisation factor applied to Pythia 6.4 distributions, as explained in Sect. 8, the comparison between Pythia 6.4 and data is meaningful only at the shape level. Tabulated values of all observed results are available in the Durham HEP database [44]. Figure 3 shows the differential cross section for $b\bar{b}$ production as a function of the dijet invariant mass. The cross section decreases with increasing mass except for a step around 550 GeV. This value corresponds approximately to twice the $p_{\text {T}}$ requirement on the leading jet, i.e. to a mass region where the flavour-creation process, with two almost back-to-back *b*-jets, becomes the dominant production mechanism. Powheg provides a very good description of the data over the whole mass spectrum, with the exception of the high-mass region where a small deficit in the prediction is seen. The MC@NLO prediction is consistently below the data for $m_{bb} <350$ GeV, at which point it becomes higher than data. This jump corresponds to the region where the flavour-creation process begins to contribute to $b\bar{b}$ production. The LO predictions (both Sherpa and Pythia 6.4) overestimate the data at low masses and underestimate them at very high masses.

The differential cross section as a function of the dijet $p_{\text {T}}$ ranges between 0.2 and $0.5 \,\mathrm {pb/\mathrm{GeV}}$, as can be seen in Fig. 4. Such a relatively constant distribution is a consequence of requiring a leading jet with $p_{\text {T}} >270$ GeV, which suppresses the flavour-creation process, which typically produces two *b*-jets with low $p_{{\mathrm T},bb}$. Without this requirement, flavour creation would overwhelm the other production mechanisms by several order of magnitudes. All MC generators provide a good description of the high-$p_{{\mathrm T},bb}$ region. Powheg and MC@NLO deviate significantly from data for $p_{{\mathrm T},bb}$ below about 200 GeV, while Sherpa overestimates the data in the region $50 \lesssim p_{{\mathrm T},bb} \lesssim 130$ GeV. Pythia 6.4 reproduces well the shape of the data.

The cross sections as a function of the azimuthal angle and of the $\eta$–$\phi$ distance between the jets are shown in Figs. 5 and 6, respectively. In these figures, the region at high angular separation is where the flavour-creation process is expected to dominate. This is visible in the peaks at $\Delta \phi \sim \pi$ and $\Delta R \sim 3$. The NLO predictions are above the data in Fig. 5 for low $\Delta \phi$ values, where the $b\bar{b}$ pair is more likely produced together with at least one other jet. They reproduce well the shape of the data distribution for $\Delta \phi \gtrsim 1$, but underestimate the cross section by a factor two in the same region. Good agreement between data and simulation with LO generators is seen. The differential cross section as a function of $\Delta R$, shown in Fig. 6, is well reproduced by Powheg. The ratio of MC@NLO predictions to the data is $\sim 0.5$ for $\Delta R \lesssim 2$, and is above the data in the intermediate $\Delta R$ region. The LO predictions do not show strong deviations from data apart from an excess for $\Delta R$ values below $\sim 0.7$.

Figures 7 and 8 show the cross sections as a function of the rapidity variables $y_B$ and $y^{*}$, respectively. The Powheg predictions reproduce well the shape of the data distribution for both observables. The LO predictions deviate from the data for $y_B$ > 1.2. The MC@NLO predictions are above the data for $y_B < 0.1$, and are significantly lower than the data for $0.3 \lesssim y_B \lesssim 1.4$, as well as for $y^{*} \lesssim 0.8$. Pythia 6.4 and Sherpa also generally describe the data well, particularly the $y^*$ distribution, although their predictions are above the data for $y_B \gtrsim 1.2$.

## Conclusion

Differential cross sections for pairs of *b*-jets have been measured in *pp* collisions at $\sqrt{s} = 7$ TeV using $\mathrm 4.2\,\text {fb}^{-1}$ of data recorded by the ATLAS detector at the LHC. Six dijet variables are investigated to probe the $b\bar{b}$ phase space: the invariant mass, the transverse momentum, and the boost of the dijet system; the azimuthal angle, the angular separation, and the rapidity difference between the two *b*-jets. The dijet system is defined as the two highest-$p_{\text {T}}$ *b*-jets in the event with $p_{\text {T}}$ > 20 GeV, $|\eta |<2.5$, requiring a minimum $\Delta R$ of 0.4. A further requirement of a jet in the event with a minimum transverse momentum of 270 GeV is applied.

The results are compared with NLO QCD predictions obtained using Powheg and MC@NLO and the LO predictions provided by Sherpa and Pythia 6.4. The use of single-jet triggers with high $p_\mathrm{T}$ thresholds significantly changes the relative weight of the different production processes with respect to an almost unbiased selection [9], with an enhancement of the gluon-splitting mechanism by strongly suppressing the low-$p_{\mathrm{T},bb}$ region where the flavour-creation process dominates. Under these conditions, MC@NLO shows significant deviations from data for all variables, both in terms of shape and normalisation. Powheg generally reproduces well the measured differential cross sections, although it underestimates the data at low $p_{{\mathrm T},bb}$. The LO predictions approximately reproduce all distributions although some bins show deviations of up to about 50%.

In general, this analysis, which is particularly sensitive to the three-jet topology, confirms that the current MC generators have significant difficulties in describing regions of phase space which are not dominated by two hard *b*-jets.

## Footnotes

1 The ATLAS reference system has the origin at the nominal interaction point. The *x*- and *y*-axes define the transverse plane, the azimuthal angle $\phi$ is measured around the beam axis, *z*, and the polar angle $\theta$ with respect to the *z*-axis. The pseudorapidity is defined as $\eta = -\ln \left[ \tan \left( \theta /2\right) \right]$ and $p_{\text {T}}$ is momentum transverse to *z*.

2 The primary vertex is defined as the vertex with the largest scalar sum of $p_{\mathrm T}^{2}$ for its associated tracks and with at least two associated tracks with $p_{\text {T}} >400$  MeV.
